# Tolerance to alkaline ambient pH in *Aspergillus nidulans* depends on the activity of ENA proteins

**DOI:** 10.1038/s41598-020-71297-z

**Published:** 2020-08-31

**Authors:** Ane Markina-Iñarrairaegui, Anja Spielvogel, Oier Etxebeste, Unai Ugalde, Eduardo A. Espeso

**Affiliations:** 1 Department of Applied Chemistry, Faculty of Chemistry, University of the Basque Country, San Sebastian, Spain; 2 Department of Cellular and Molecular Medicine, Centro de Investigaciones Biológicas Margarita Salas, C.S.I.C., Ramiro de Maeztu, 9, 28040 Madrid, Spain

**Keywords:** Fungal genes, Cellular microbiology

## Abstract

Tolerance of microorganisms to abiotic stress is enabled by regulatory mechanisms that coordinate the expression and activity of resistance genes. Alkalinity and high salt concentrations are major environmental physicochemical stresses. Here, we analyzed the roles of sodium-extrusion family (ENA) transporters EnaA, EnaB and EnaC in the response to these stress conditions in the filamentous fungus *Aspergillus nidulans*. While EnaC has a minor role, EnaB is a key element for tolerance to Na^+^ and Li^+^ toxicity. Adaptation to alkaline pH requires the concerted action of EnaB with EnaA. Accordingly, expression of *enaA* and *enaB* was induced by Na^+^, Li^+^ and pH 8. These expression patterns are altered in a *sltA*Δ background and completely inhibited in a mutant expressing non-functional PacC protein (*palH72*). However, a constitutively active PacC form was not sufficient to restore maximum *enaA* expression. In agreement with their predicted role as membrane ATPases, EnaA localized to the plasma membrane while EnaB accumulated at structures resembling the endoplasmic reticulum. Overall, results suggest different PacC- and SltA-dependent roles for EnaB in pH and salt homeostasis, acting in coordination with EnaA at pH 8 but independently under salt stress.

## Introduction

Ion homeostasis is an essential biochemical process for cell life influencing a wide range of cellular functions from osmotic regulation to enzyme activity. In addition, certain ions, such as sodium, calcium, and lithium become toxic at a high cytoplasmic concentration^1^. Therefore, microorganisms have developed specific transport mechanisms to maintain an adequate intra and extracellular distribution of certain ions. Examples are the maintenance of low cytoplasmic Na^+^/K^+^ ratio and the extremely low cytoplasmic concentrations of Li^+^ (< 10^–7^ M) and Ca^2+^ (aprox. 10^–7^ M)^2–4^. Modulation of cytoplasmic cation contents is mainly achieved by active transmembrane transporters that facilitate efflux and influx of ions through the plasma membrane or by an efficient storage of ions in intracellular compartments such as vacuoles^5^. In addition, the activity of plasma-membrane transporters generates an electrochemical gradient across the membrane which stores energy that can be used by other transport mechanisms acting as antiporters and/or symporters. In this respect, the transmembrane electrochemical proton gradient has a major influence on the transmembrane potential and on the functionality of ion transport across the plasma membrane in both directions^6^. But, most importantly, proton concentration determines the pH value and is the basis of a major abiotic stress/stimulus for the cell^7^.

Filamentous fungi are able to grow over a wide pH range with almost constant intracellular pH and transmembrane potential of approximately − 200 mV^8,9^. A change in the extracellular pH, either towards acidification or to alkalinization, is an important stimulus to a cell or organism. This might cause alterations in the electrochemical gradient and its maintenance, with direct impact on the bidirectional transport of solutes and other compounds. Changes in extracellular pH also influence the activity of secreted enzymes and metabolites, most of which have an optimal pH for maximal activity. Major adaptation processes occur, mainly altering the expression pattern of genes that are important for ion homeostasis and whose products are pH regulated^10^.

Regarding transport mechanisms, intracellular levels of Na^+^/K^+^ are maintained at neutral and acidic pH by Na^+^/H^+^ and K^+^/H^+^ antiporters. In *S. cerevisiae* intracellular low sodium maintenance relies on the Nha1p Na^+^/H^+^ antiporter which is able to extrude Na^+^, K^+^, and Li^+^^11^. These antiporters depend on the transmembrane ∆pH, being less functional when the external pH is higher than the cytoplasmic pH, resulting in a decreased pH gradient. The intracellular pH value of *Aspergillus spp.* is approximately 7.6^8^, and when the external pH turns alkaline, transporters are required that can act independently of an external proton gradient. In most cases, P-type Ena ATPases mediate Na^+^/K^+^ transport under alkaline conditions^5^.

Ena ATPases have been identified in bryophytes, protozoa and fungi^12–16^. Furthermore, it has been shown that fungal Ena-like ATPases display also transport activities for Li^+^ and Rb^+^ in addition to Na^+^ and K^+^^5^. Most extensive studies are available for *Saccharomyces cerevisiae* Ena ATPases. However, this Saccharomycete is an acidophilic organism and first results from human pathogenic *Candida* species and the plant pathogen *Ustilago maydis* point to major differences in the activity and regulation of these transporters^17^. It is worth noting the fact that pathogenic microorganisms might have adapted to the high intracellular potassium ion concentrations of their hosts, being a cause of evolutionary specialization. In the case of *Aspergillus nidulans*, EnaA (AN6642) was first reported by Han and colleagues^18^ in connection with the high osmolarity glycerol pathway (HOG). These authors observed a transcriptional up-regulation pattern of a plasma membrane ATPase similar to the members of the HOG pathway, dependent on the presence of high NaCl concentration. However, induction of *enaA* was not observed when heat or oxidative stress was applied^18^ or to be subjected to the control of the bZIP transcription factor AtfA, a key element in the coordination of environmental stress response including oxidative stress^19^.

Three major regulatory pathways mediate tolerance of *A. nidulans* to alkalinity. Ambient pH response is mediated by the well-known Pal/PacC pathway^20^. The signalling pathway comprises six so called Pal proteins (PalA, PalB, PalC, PalF, PalH, and PalI) and transduces the alkaline-pH signal into the cell to its final acceptor, the zinc-finger transcription factor PacC. At neutral-to-alkaline ambient pH conditions PacC is activated through two proteolytic steps, being the first modification dependent on the Pal pathway. The final 27 kDa processed from of PacC acts as an activator of alkaline-expressed genes and as a repressor of acid-expressed genes^20,21^. The role of CrzA and SltA transcription factors in cation homeostasis and alkaline pH regulation has been determined^16,22,23^. In *S. cerevisiae,* expression of *ena* genes requires the integrity of the PacC/Pal homologous RIM pathway^5^. ScCrz1p, the CrzA orthologue, is one major regulator of ScEna1p^24,25^. To date, in *A. nidulans*, regulation of *ena* genes has not been addressed in detail. Expression of *enaA* is not regulated by CrzA, and SltA was shown to play a minor effect on *enaA* transcription^16^.

In this study, we focus on the functional analysis of the three ScEna1p orthologues of *Aspergillus nidulans,* EnaA, EnaB and EnaC^16,18^. Tolerance to extracellular excess of sodium and lithium at low or high pH has been evaluated in strains carrying all possible combinations of null *ena* alleles. Transcriptional regulation of *ena* genes and the subcellular localization of EnaA and EnaB has been explored in growth conditions combining excess of Na^+^/Li^+^ and alkalinity, as well as the involvement of ambient pH regulatory pathway mediated by PacC and the cation stress responsive pathway mediated by SltA as possible regulatory mechanisms. Results suggest a key role of EnaB in cation homeostasis and a concerted action with EnaA in the response to alkaline pH.

## Results

### Genes coding for ENA-like proteins in *A. nidulans*

**Figure 1:**
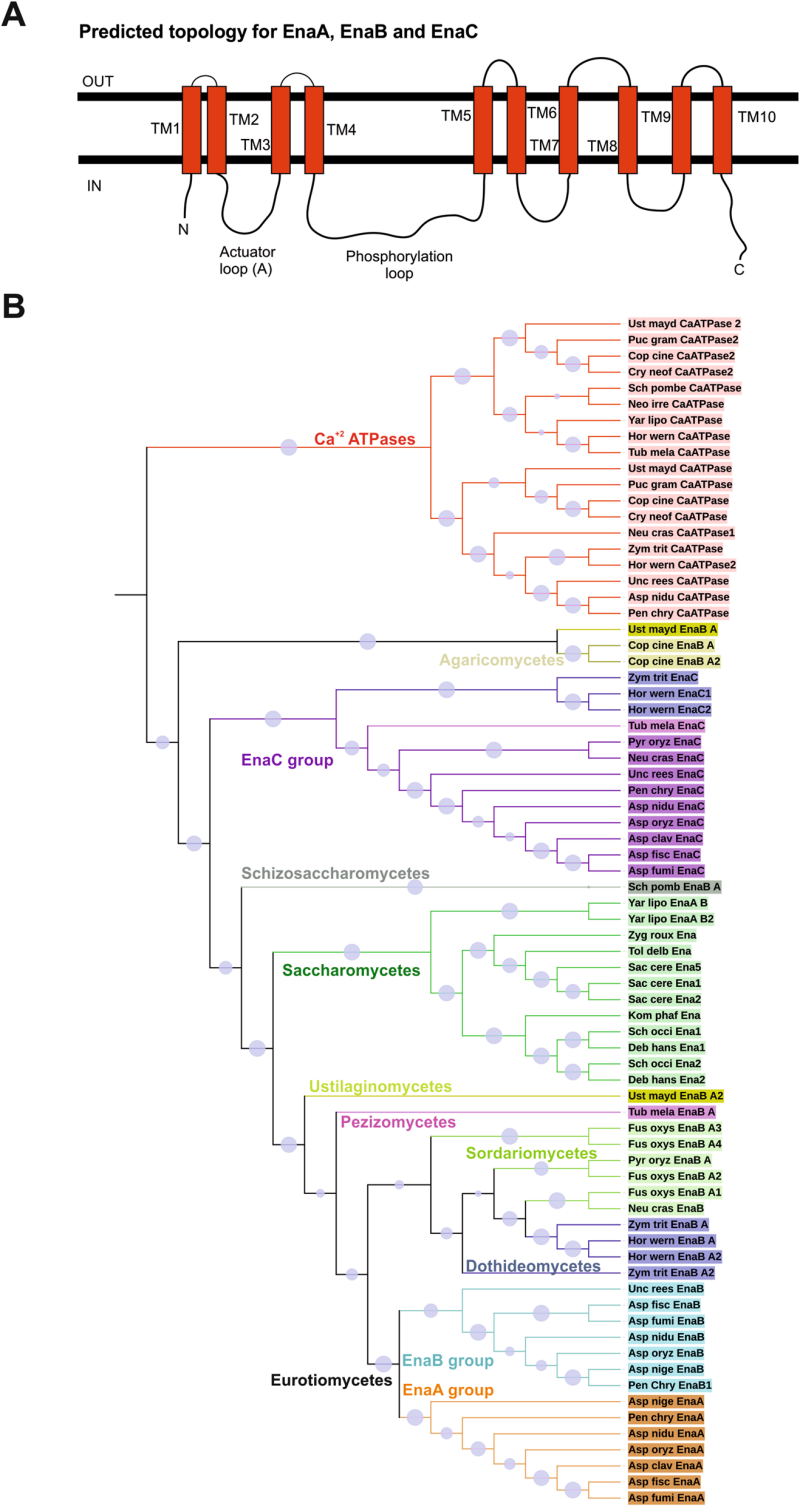
ENA-like proteins in *Aspergillus nidulans*. (**A**) Proposed two-dimensional model of EnaA and EnaB describing the predicted membrane topology and principal conserved functional domains among P-type ATPases. (**B**) Maximum-likelihood tree of Na^+^, K^+^, H^+^ ATPases from fungi and yeast. EnaA and EnaB clustered in the same group of ATPases while EnaC clearly differentiated and belong to a separate clade. MEGA7 software was used to generate the tree^58^ and it was edited with iTOL^59^. Blue circles in each clade represent bootstrap values. Species; Asp_clav: *Aspergillus clavatus*; Asp_fisc: *Aspergillus fischeri*; Asp_fumi: *Aspergillus fumigatus*; Asp_nidu: *Aspergillus nidulans*; Asp_nige: *Aspergillus niger*; Asp_oryz: *Aspergillus oryzae*; Deb_hans: *Debariomyces hansenii*; Fus_oxys: *Fusarium oxysporum*; Hor_wern: *Hortaea werneckii*; Kom_pahf: *Komagataella phafii*; Neu_cras: *Neurospora crassa*; Pen_chry_ *Penicillium chrysogenum*; Pyr_oryz_ *Pyricularia oryzae*; Sac_cere: *Saccharomyces cerevisiae*; Sch_occi: *Schwanniomyces occidentalis*; Tol_delb: *Torulaspora delbrueckii*; Unc_rees: *Uncinocarpus reesii*; Ust_mayd: *Ustilago Maydis*; Zyg_roux: *Zygosaccharomyces rouxii.* Cop_cine: *Coprinopsis cinerea*; Cry_neof: *Cryptococcus neoformans*; Puc_gram: *Puccinia graminis*; Tub_mela: *Tuber melanosporum*; Yar_lipo: *Yarrowia lipolytica*; Sch_pomb: *Schizosaccharomyces pombe*; Neo_irre: *Neolecta irregularis*; Zym_trit: *Zymoseptoria tritici*.

As previously noted by Spielvogel and collaborators^16^, the genome of *A. nidulans* contains three genes coding for putative ENA proteins which were designated *enaA *(*AN6642*), *enaB *(*AN1628*) and *enaC *(*AN10982*). A multiple alignment of An-ENA sequences including ScEna1p is shown in Supplementary Fig. 1. EnaA, EnaB and EnaC show high conservation with ScEna1p (YDR040C) sequence (65%, 67% and 59%, respectively). Nevertheless, the three An-ENA proteins clustered in one of the eight clades that allocate the 22 ATPases identified in *A. nidulans* (Supplementary Fig. 2A)^26^. Conserved domains (Pfam domains) are found among ENA homologues denoting their roles as P-type ATPases, such as (I) cation-ATPase-N (pfam00690), (II) E1–E2 ATPase (pfam00122), (III) haloacid dehalogenase-like hydrolyase (HAD) (COG4087. pfam00702) and (IV) cation-ATPase-C (pfam00689) (Supplementary Fig. 2B). A more detailed analysis of *A. nidulans* ENA protein sequences compared to ScENA1p showed the presence of several small motifs characteristic of this family of transporters, such as the Actuator domain (TGES^183^), the Mg^2+^ binding motif (DGVND^761^), and sequences belonging to the catalytic site of P-type ATPases: the signature sequences DKTGT^393^, TGD^675^ and DPPR^652^ and residues of the nucleotide binding domain (F^537^, K^542^, K^561^) (indicated coordinates as in ScEna1p, Supplementary Fig. 1)^15,27^. Distribution of these functional motifs was also conserved among the An-ENA ATPases (Supplementary Fig. 1). As for ScEna1p P-type ATPases, An-ENA transporters were predicted to harbour 10 putative transmembrane spanning regions (TMHMM and HMMTOP predictions) with N- and C-terminal regions being directed to the cytoplasmic side if located at the plasma membrane (Fig. 1A).

Additional in silico searches showed the presence of at least one and up to four ENA-like ATPase coding genes in Saccharomycetes and filamentous fungal genomes (Fig. 1B). The phylogram, based on the amino acid sequence of putative and described Na^+^, K^+^, H^+^ ATPases, revealed that EnaA, EnaB, and EnaC allocated in two different clades. While the EnaA and EnaB groups clustered, EnaC was included into a separate group of ATPases. EnaC shared similarities with HwEna1 and HwEna2 of *Hortaea werneckii*, which have been described to constitute a new family of ENA-like ATPases, although their functions are not completely determined yet^28^. Of interest is the fact that Ena P-type ATPases from Saccharomycetes constituted a monophyletic clade in this phylogenetic analysis.

In summary, fungal genomes encode several ENA-like ATPases (2–4) showing significant conservation in their sequences and composition of functional domains. However, specific transport activities as well as regulatory mechanisms or alternative functions must be determined experimentally and not only inferred from in silico predictions.

### Functional analyses of AnENA transporters

**Figure 2:**
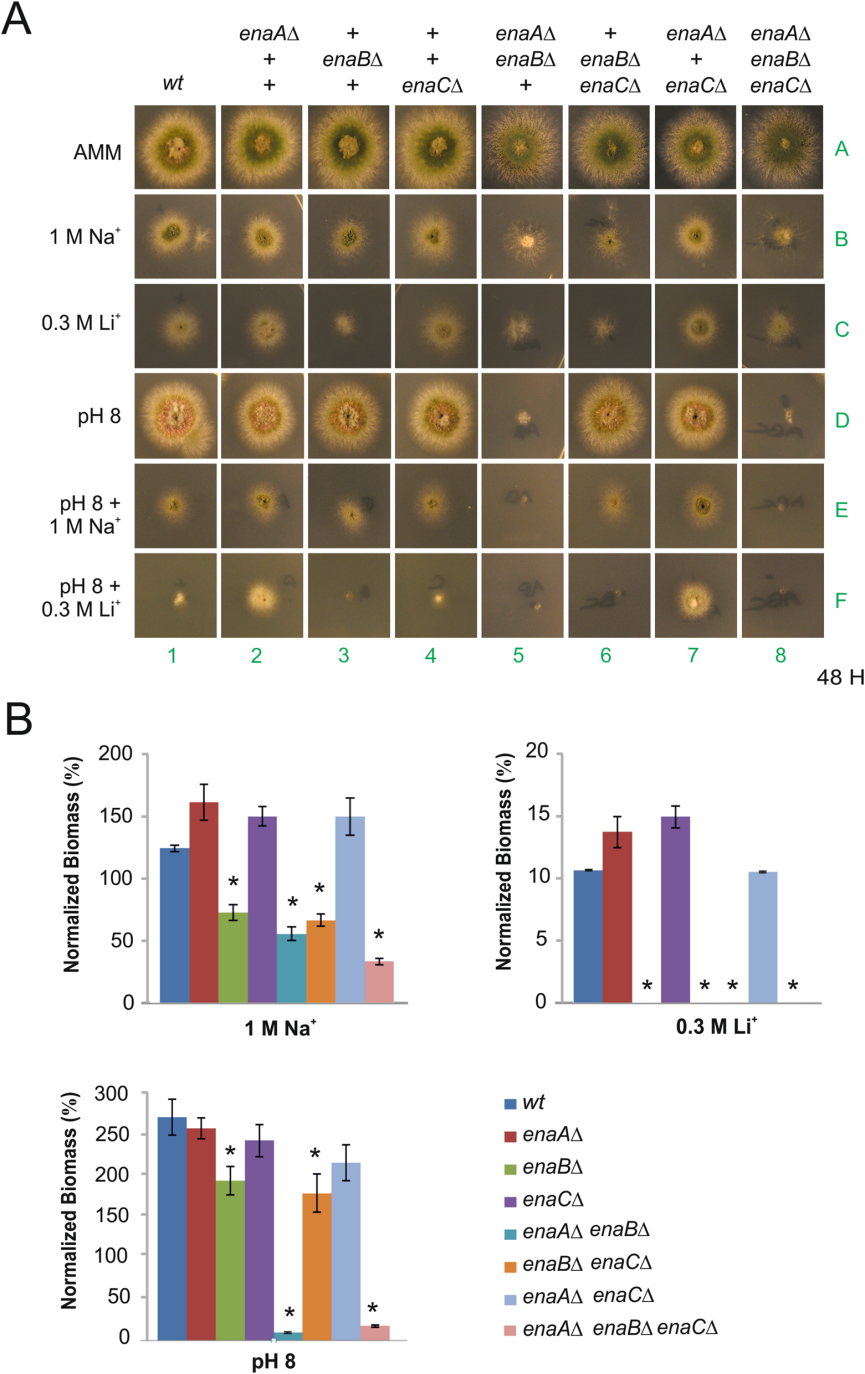
Functional analysis of null *ena* mutants. (**A**) Spores of single-, double- and triple-null strains were point-inoculated and images of colonies were taken after 48 h of incubation at 37 °C in solid AMM (row 1), AMM supplemented with 1 M NaCl (Na^+^; row 2) or 0.3 M LiCl (Li^+^; row 3), AMM adjusted to pH 8 with 50 mM Tris–HCl pH8 (alkaline pH; row 4), and AMM that combined the Tris–HCl buffer with either 1 M NaCl (pH 8 + Na^+^; row 5) or 0.3 M LiCl (pH 8 + Li^+^; row 6). (**B**) Biomass production was determined by measuring the dry weight of cells grown in liquid AMM for 24 h at 37 °C with and without elevated concentration of cations (1.0 M NaCl and 0.3 M LiCl) or alkaline pH. Data is presented as percentages and normalized by designating the growth of each strain in standard AMM (no stress agents) as 100%. Graphs show the means of three replicates per strain and condition, and error bars indicate standard deviation. Asterisks indicate a significant variation in biomass production (p < 0.05) as compared to the reference wild-type strain. N = 3.

To determine a functional role for An-ENA orthologues, single, double and triple knock-outs of *enaA*, *enaB*, and *enaC* were constructed by gene replacement and subsequent meiotic recombination to obtain all possible mutant combinations (see list of strains in Supplementary Table 1). Single, double and the triple null *ena* mutant colonies grew on solid standard *Aspergillus* minimal medium (AMM) showing no appreciable alterations on radial growth or conidiation (Fig. 2A, see AMM, row A). Sensitivity of *ena* mutant strains to an elevated extracellular concentration of sodium (1.0 M) or lithium (0.3 M) was tested (Fig. 2A, rows B and C, respectively). When each the three-single knock-out mutants were grown in the presence of 0.3 M Li^+^ and 1.0 M of Na^+^ only *enaB*∆ showed a severe growth inhibition with the former (only a minor effect was detected with Na^+^). The excess of Li^+^ also affected all double and triple null mutants carrying the null *enaB* allele. A stronger sensitivity to Na^+^ was also observed in double mutants containing *enaB*∆ and the triple mutant. In contrast, the double *enaA*∆ *enaC*∆ mutant showed a level of tolerance to sodium comparable to that of wild-type strain. These results identified EnaB as the principal element in tolerance to Na^+^ and Li^+^ stress.

Since alkalinity is a signal inducing expression of *enaA* gene (see references^16,18^, and below), tolerance of *ena* null mutant strains to alkaline pH was also analyzed (Fig. 2A, row D). Single null *ena* strains grew as well as the wild-type strain at pH 8. However, double deletion of *enaA* and *enaB* resulted in strong sensitivity. Other double null mutant combinations displayed normal colonial growth. As expected, the triple null mutant displayed a pH 8-sensitive phenotype comparable to that displayed by the double *enaA*∆ *enaB*∆ strain. These results indicated that activities of EnaA and EnaB are jointly required for tolerance to ambient alkalinity.

Sensitivity of null *ena* strains to high concentrations of Na^+^ and Li^+^ was also tested at alkaline pH (Fig. 2A, rows E and F). Besides the expected growth inhibition of strains lacking both EnaA and EnaB activities, a negative effect of combining alkalinity and high Na^+^ concentration was not observed in the other null *ena* mutant strains. When alkaline pH and elevated Li^+^ concentration were combined, growth of the wild-type strain was largely impaired. In this growth condition, the null *enaC* strain showed a wild-type phenotype and growth of the *enaB*∆ and *enaB*∆ *enaC*∆ strains were inhibited. In line with earlier observations, double *enaA*∆ *enaB*∆ and the triple *ena* mutants were sensitive to alkalinity. Interestingly the single *enaA*∆ strain showed a characteristic compact phenotype which was also observed for the *enaA*∆ *enaC*∆ mutant. These results indicate that EnaB has an important role in Li^+^ tolerance especially under alkaline conditions, even contributing to increased tolerance when EnaA function is missing.

Determination of mycelial mass growth in liquid medium (Fig. 2B) confirmed the important role of EnaB in tolerance to excess of Na^+^ and Li^+^. Meanwhile cultures of strains having the EnaB function showed comparable levels of mycelial mass in the presence of 1.0 M Na^+^, deletion of *enaB* reduced fungal growth to almost 50% (Fig. 2B). Deletion of *enaB* had a greater effect in liquid medium containing 0.3 M Li^+^, with the strongest growth inhibition observed. Mycelial masses measured at alkaline-pH also showed a significant reduction in the absence of EnaB activity in single and double null *enaB enaC* mutants, but this effect was more severe when EnaA activity was lost, as shown before on solid minimal medium. In summary, EnaA and EnaB have a principal role in adaptation to an alkaline pH environment. But EnaB function seems to be required also for Na^+^/Li^+^ adaptation and is crucial for Li^+^ homeostasis at alkaline pH.

### Transcriptional regulation of *ena* genes

**Figure 3:**
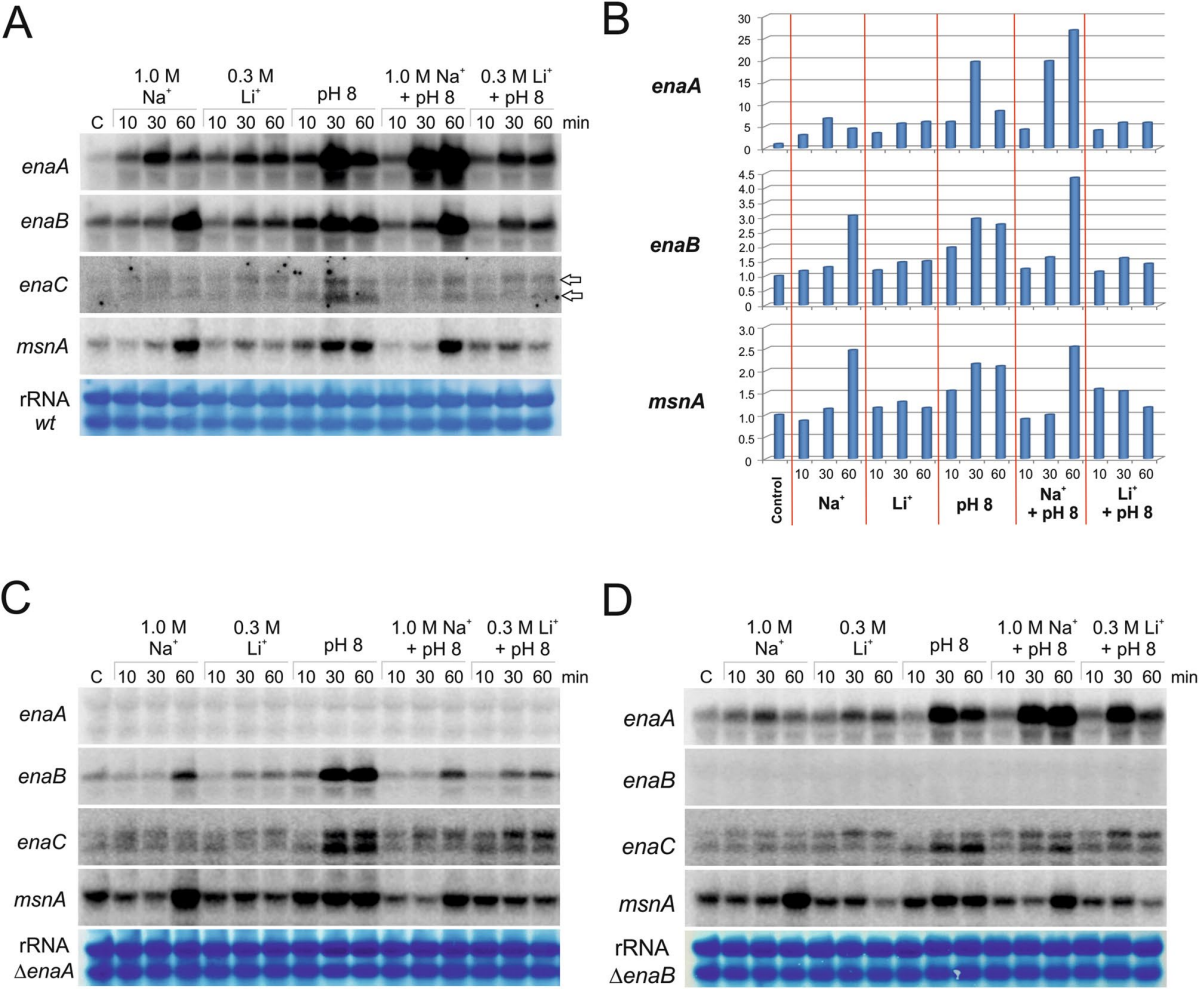
Expression analysis of *ena* genes. Northern-blot analyses showing transcript levels of *enaA, enaB, enaC* and *msnA* in samples grown in AMM for 18 h (lane C) and subsequently cultivated with 1 M NaCl (Na^+^), 0.3 M LiCl (Li^+^), at pH 8 or in AMM that combined pH 8 with either 1.0 M NaCl (pH8 + Na^+^) or 0.3 M LiCl (pH8 + Li^+^) for additional 10, 30 or 60 min. (**A**) and (**B**) show expression levels in a wild-type strain and their quantification, while (**C**) and (**D**) show the results corresponding to null *enaA* and null *enaB* backgrounds, respectively. Ribosomal RNA (rRNA) stained with methylene blue was used as a loading control. The graphs in (**B**) show the ratios between average pixel intensity for each hybridization band and the corresponding loading control band. Average pixel intensity for each band was quantified using Image J software (version 4.0; Fujifilm, Valhalla, NY).

Series of Northern blot analyses were conducted to analyze the regulation of expression of *ena* genes in total RNAs isolated from mycelia grown at alkaline pH, in excess of Na^+^ or Li^+^, and the mixture of high levels of either Na^+^ or Li^+^ with alkalinity. In agreement with previous results from Northern analyses^16,18^ and RNA sequencing^10^, we confirmed that the expression of both *enaA* and *enaB* is regulated by these stresses. Under non stressing conditions (C, control condition), expression of *enaA* was detected and a positive effect was observed after addition of large amounts of Na^+^ or Li^+^, or by alkalinizing the cultures (Fig. 3A). Addition of 1.0 M Na^+^ or 0.3 M Li^+^ resulted in a moderate increase of *enaA* mRNA levels, sixfold and fivefold change, respectively, but a stronger effect, 19-fold change, was observed in response to alkaline pH, with a noticeable peak of expression at 30 min (Fig. 3B). Yet, when elevated Na^+^ concentrations were combined with pH 8, transcript levels continued increasing throughout the experiment (60 min) showing a 27-fold change with respect to initial values. However, this synergic response was not observed when combining alkalinity and 0.3 M Li^+^. Despite the fact that reads mapping at the *enaA locus* in our RNA-seq experiments^10^ suggest the existence of alternative splicing events at the three introns predicted in the coding region (i.e. the processed form seems to be more abundant in the wild-type at pH 8 than under standard culture conditions), Northern hybridization did not indicate the generation of transcripts of different sizes (Fig. 3A).

Northern blot in Fig. 3A, and its quantification in 3B, shows that *enaB* transcript was also detected under non stressing conditions*.* Variation of transcript abundance of *enaB* and *enaA* genes seemed to follow a comparable trend, but with lower intensity in the case of *enaB*, probably due to higher levels of expression under non-stressing conditions, compared to *enaA*. Even so, differences were observed principally regarding Na^+^ stress, which induced a continuous increase in *enaB* expression through 1 h of treatment. As previously noted for *enaA*, combination of high Na^+^ and alkaline pH resulted in a higher expression than singly implemented stress conditions. It is worth mentioning that *enaB* mRNA levels remained almost unchanged after addition of 0.3 M Li^+^, which contrasts with the severity of the growth defect shown by the null *enaB* strain under these stress conditions but on solid medium.

**Figure 4:**
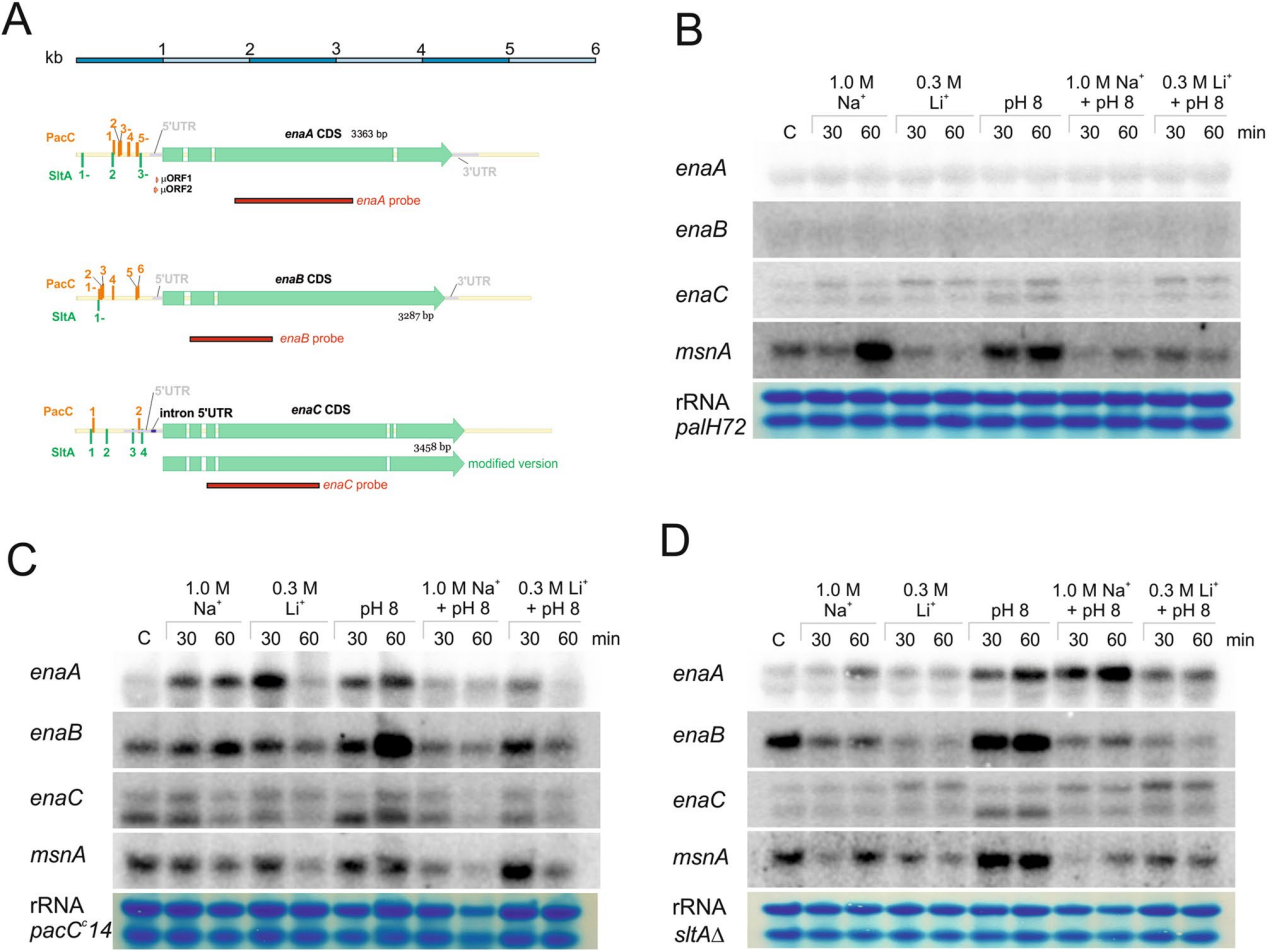
Transcriptional regulation of *ena* genes by PacC and SltA. (**A**) Schematic representation of *enaA, enaB* and *enaC loci* based on the information available in the AspGD database and our RNA-seq results. At the top a scale of 6 kb is shown. The last intron of *enaC* is not processed and adds 13 codons to the coding sequence (see main text). Alternative splicing events are also likely in *enaA* and mainly *enaC* (see main text). The promoter regions of *ena* genes are included, with the number and distribution of PacC and SltA binding sites. The red bars represent the radioactive probes used in Northern-blot experiments. (**B**) Northern-blot analyses showing transcript levels of *enaA, enaB, enaC* and *msnA* in samples collected in the absence, C, and presence, 30 or 60 min, of high cation concentrations (same as in Fig. 3). (**B**) Corresponds to the *palH72* background, (**C**) to a *pacC*^*c*^*14* background and (**D**) to a null *sltA* background. Ribosomal RNA (rRNA) stained with methylene blue was used as a loading control.

In the case of *enaC*, Northern blot analyses detected two possible transcripts for this gene (indicated with arrows, right side Fig. 3A), being the high mobility band slightly induced by the presence of alkaline pH. The size of the two hybridization signals did not coincide with those of *enaA* or *enaB* transcripts, and thus, a cross-hybridization phenomenon was excluded. The presence of two transcripts may be a consequence of possible alternative splicing events at introns 1 and 3 of the coding region, as shown by our RNA-seq data. Furthermore, the last intron of *enaC* is not processed, adding 13 codons to the sequence but without altering the reading frame. The corresponding set of 13 amino acids is part of the fifth transmembrane domain. The corrected EnaC sequence, including the additional 13 amino acids, was used in the analysis shown in Fig. 1 and Supplementary Fig. 1 (see also below in Fig. 4A). However, these 39 nucleotides do not explain the difference in size observed between both *enaC* transcripts. Considering that multiple RNA-seq reads map to the intergenic region for *enaC* and *An7665* (not shown), and that this region would include an additional target site for PacC and two sites for SltA, it is tempting to suggest that the presence of two transcription start sites could account for the existence of two *enaC* transcripts.

MsnA is a transcription factor involved in saline stress response^18^. Figure 3A shows expression levels of *msnA* following a similar pattern to that described before for *enaB*. High Na^+^ elevated the expression of *msnA* (Fig. 3A,B). In contrast, high Li^+^ had no effect or even we detected a negative effect of this cation by preventing activation of *msnA* expression in the presence of alkaline pH. These results suggest a common transcriptional regulatory mechanism acting on *msnA* and *enaB* genes, and probably on *enaA*.

To further investigate a possible influence of Ena proteins in their own transcriptional regulation, expression of the remaining *ena* genes was studied in single knock-out mutants *enaA*Δ and *enaB*Δ. Figure 3C shows that the temporal profile of *enaB*, *enaC* and *msnA* did not significantly change in the absence of *enaA.* Likewise, in the absence of *enaB* (Fig. 3D), no major differences were observed in *enaC* and *msnA* gene regulation*,* whereas the response of *enaA* to the combination of alkalinity and Li^+^ resulted in a stronger early (maybe compensatory) response (30 min) compared to samples from cells where *enaB* is present.

Taken together, these results indicate that separately Na^+^, Li^+^ and alkaline pH may induce *enaA* and *enaB* expression at different levels. However, a combination of high Na^+^ and alkalinity caused the greatest levels of expression for these genes, suggesting a major need of these ATPases under both stresses. Interestingly, the presence of lithium prevented the synergic effect of alkaline pH, indicating the presence of differential regulatory mechanisms acting under each cation stress.

### Regulation of *ena* genes by PacC and SltA transcription factors

Since Na^+^ and Li^+^ cations, alkalinity and its combination differently modulate *enaA* and *enaB* expression, the role in *ena* expression of two transcription factors mediating response to ambient pH and cation stresses, PacC and SltA, was monitored^16,20^.

To study the role of PacC on the regulation of *ena* genes, we analyzed their expression levels in two opposed ambient-pH mutant backgrounds. A mutant with a null *palH72* mutation prevents proteolytic processing of the primary translational form of PacC, PacC72kDa, disabling functionality^29^. On the other hand, the *pacC*^*c*^*14* mutation, constitutively generates the active 27 kDa version of PacC, at any ambient pH^30^. Northern analyses showed the strict requirement of proper PacC signalling for *enaA* and *enaB* expression (Fig. 4B). Transcripts of *enaA* and *enaB* were not detected in the null *palH72* background under any condition (Fig. 4B). In contrast, detection of putative *enaC* transcripts and expression of *msnA* remained essentially as described in a wild-type PacC background (compare with Fig. 3A).

The constitutive activation of PacC did not result in a pH-independent expression of *ena* genes. Expression of *enaA* in the *pacC*^*c*^*14* mutant required addition of Na^+^ or ambient pH alkalinisation, but the expected synergic effect by combining both stresses was not observed (Fig. 4C). Excess of extracellular Li^+^ had a partial effect and *enaA* expression was high at 30 min but strongly inhibited at 60 min. A similar effect was observed in samples treated with Li^+^ and alkaline pH. In contrast, the *enaB* transcript showed almost constant levels under most conditions tested, but a strong transcriptional response after alkaline stress induction was still observed at 60 min. A negative effect of Li^+^ on *enaB* expression was observed at 60 min, with or without media alkalinisation, and also, the mixture of Na^+^ and alkaline pH did not induce the expected positive effect. Transcript levels of *enaC* and *msnA* followed similar patterns to those described in the wild-type or *palH72* backgrounds. These results suggest the existence of two additional regulatory mechanisms with opposite effects: a positive alkaline-pH dependent effect and a negative high-Na^+^ modulation.

To determine the role of SltA on their regulation, the expression profiles of *ena* genes were analyzed in a *sltA*∆ mutant strain under the above stress conditions. Figure 4D shows that the expression pattern of *enaA* in the null *sltA* strain was similar to that described in the wild-type background. Absence of *sltA* resulted in higher basal *enaB* transcript levels which were increased at alkaline pH but, as mentioned above, a negative effect was also detected when high concentrations of Na^+^ or Li^+^ were added to cultures. The expression pattern of *msnA* nearly paralleled that of *enaB*.

In agreement with possible direct roles of PacC and SltA on the regulation of *enaA* and *enaB* genes, we found putative target sites for these transcription factors in their promoters. Searches confirmed the presence of five consensus PacC (5′-GCCAAG-3′) and three SltA (5′-AGGCA-3′) sites in *enaA* promoter (1 kb upstream of ATG). Six PacC and one SltA target sequences in *enaB* promoter and two PacC and four SltA sites in *enaC* promoter (Fig. 4A). Number and locations of PacC sites in *enaA* and *enaB* promoters are in accordance with a direct role for this TF, as has been demonstrated before for other genes (see discussion). Scattering of SltA sites might be a cause for the reduced role of this TF in their regulation.

### Expression profiles of EnaA and EnaB proteins under stress conditions

**Figure 5:**
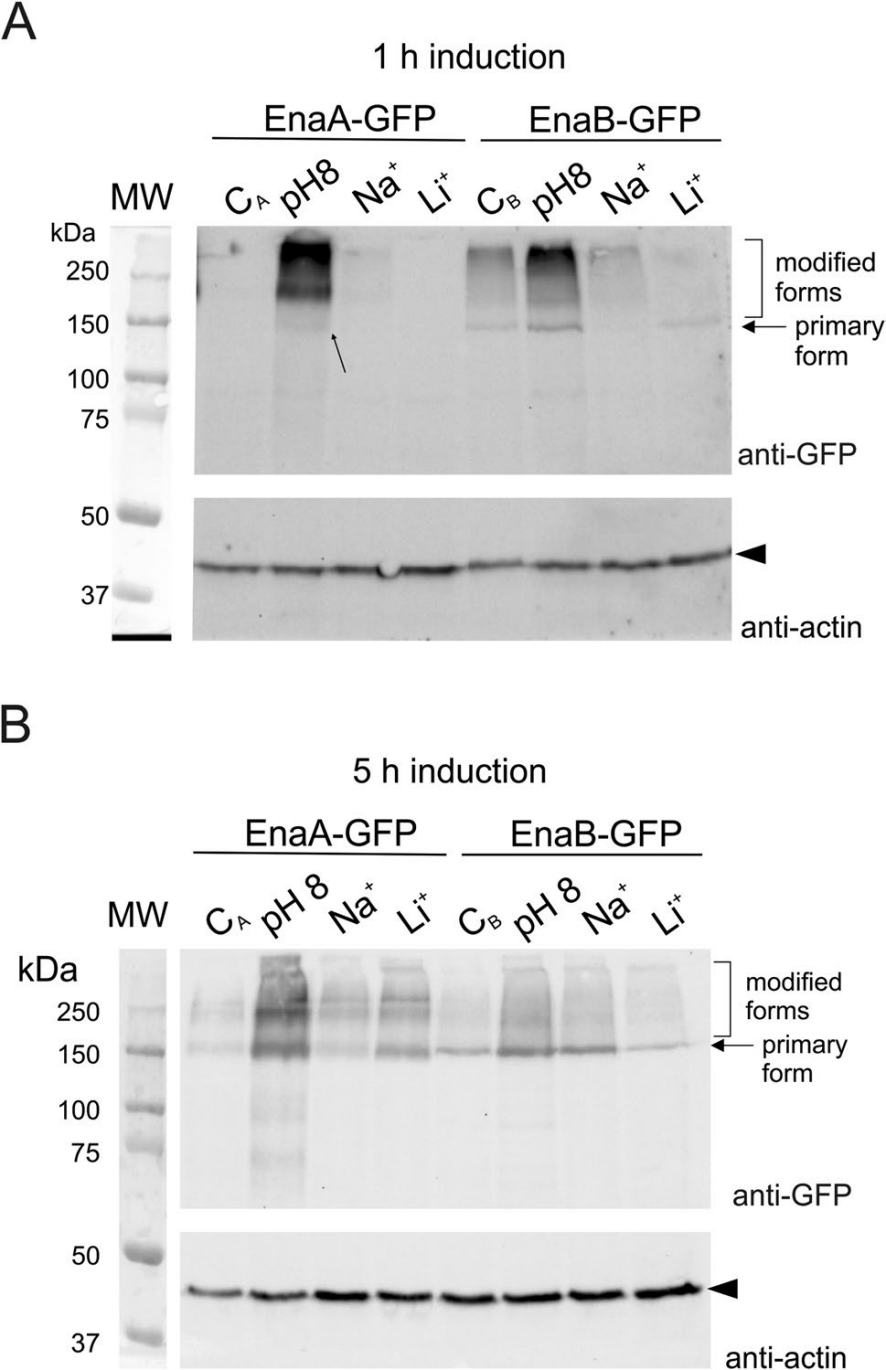
Immunodetection of EnaA-GFP and EnaB-GFP. Western-blot analyses showing patterns of EnaA and EnaB bands in total protein extracts of mycelia cultured in the absence (C) and presence of 1 M NaCl, 0.3 M LiCl or alkaline pH for (**A**) 1 h and (**B**) 5 h. GFP-tagged fusion were subjected to immunodetection using α-GFP primary antibody. The expected full-length chimeras are indicated with an arrow and the detected modified forms are indicated with a bracket. Actin was used as loading control.

To further analyze EnaA and EnaB function, we constructed strains expressing C-terminally GFP-tagged versions. These strains showed a wild-type phenotype in all conditions tested (not shown), indicating that the chimaeras did not cause any dysfunction to the ENA system. Both EnaA-GFP and EnaB-GFP were detected at very low levels in mycelia grown in AMM without Na^+^/Li^+^ or alkaline pH stresses, in line with Northern blot results. Immunodetection experiments further confirmed barely detected EnaA-GFP (with expected size of 150 kDa) in protein extracts from mycelia grown for 19 h and 24 h in standard AMM (C_A_ in Fig. 5A,B, respectively). In the case of EnaB-GFP, and in agreement with higher levels of *enaB* transcript under basal AMM conditions, we detected a band with the expected size of the full chimera, nearly 150 kDa (see arrow in lanes C_B_, Fig. 5A,B). We also perceived a smear of low mobility bands starting at the limit of the SDS polyacrylamide resolution gel (indicated with a square bracket). All these forms, primary and low mobility, became more evident when protein extracts of mycelia were subjected to alkaline pH stress (Fig. 5). These dispersed high-molecular weight species of fusion proteins might reflect post-translationally modified forms. EnaA-GFP and EnaB-GFP were expressed early in alkaline medium compared to the effect of adding Na^+^ or Li^+^ (compare the relative band intensities of EnaA-GFP and EnaB-GFP in Fig. 5A,B). However, EnaB-GFP was expressed under standard culture conditions and although alkaline pH induced the strongest response, the chimera was also detected 1 h after Na^+^ and Li^+^ treatments. Overall, results of immunodetection experiments suggest that compared to pH 8, an increase in the concentration of both proteins under Na^+^ or Li^+^ stress conditions is delayed.

### Subcellular localization of EnaA and EnaB

**Figure 6:**
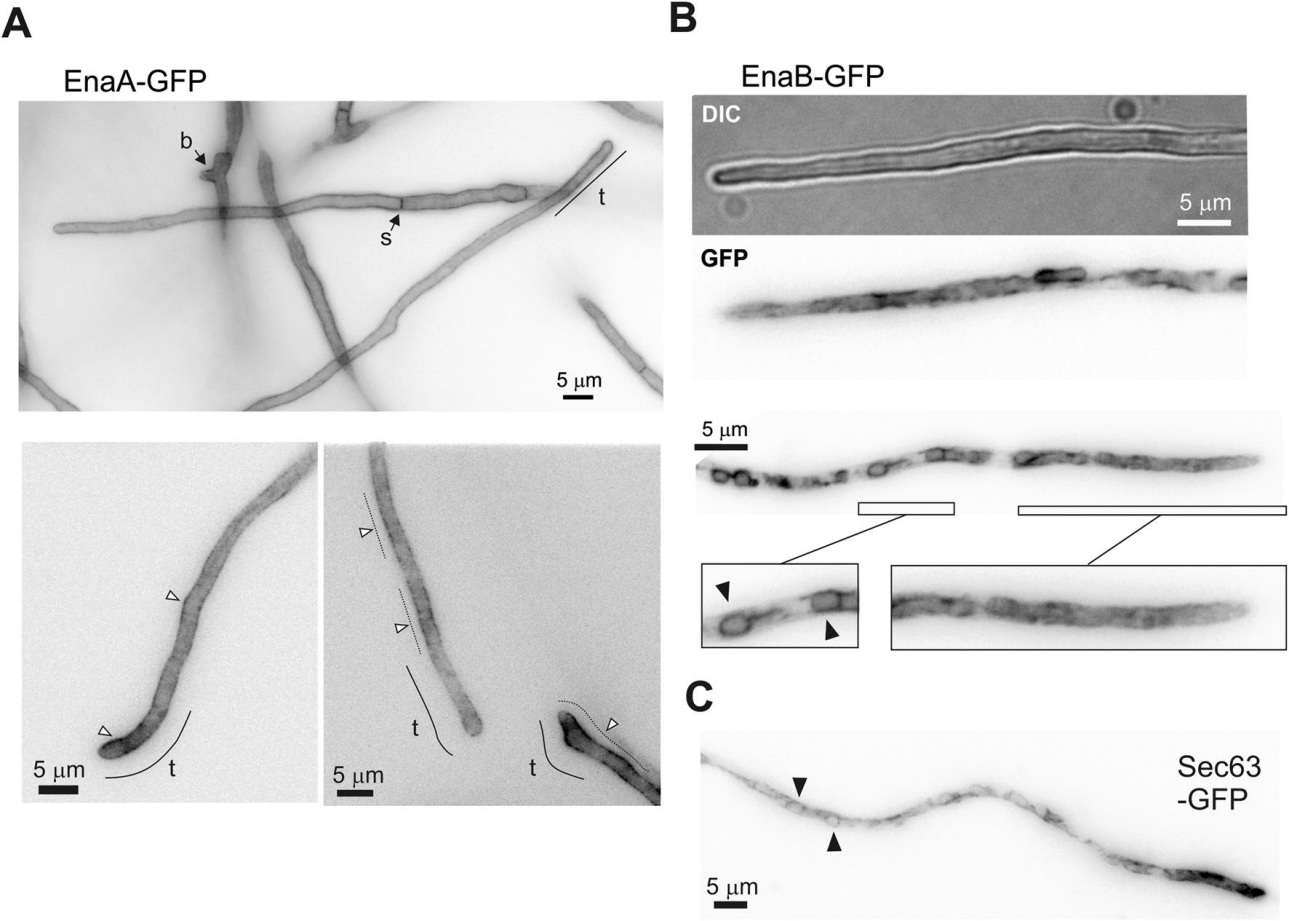
Subcellular localization of EnaA-GFP and EnaB-GFP. Cells were grown in selective WMM for 16 h at 25 °C and shifted to freshly prepared WMM adjusted to pH 8 with 0.1 M Na_2_HPO_4_. Images were taken approximately 1.5 h after the shift. (**A**) Localization of EnaA along the plasma membrane; in the tip (t), brunches (b), septa (s) and grouped in clusters (white arrowheads). (**B**) Localization of EnaB in internal membranous organelles. Magnifications correspond to the tip region, where EnaB distributes in a network of strands and tubules, and a more distal region where is localized in ring-shaped structures (black arrowheads). Localization of Sec63-GFP is included as an ER marker. Green fluorescence images are shown in inverted gray contrast. Bars = 5 μm.

EnaA and EnaB are expected to be transmembrane proteins. In agreement with previous protein expression results, cells from *enaA*-*gfp* and *enaB*-*gfp* transformants showed no fluorescence in standard microscopy medium (WMM; not shown). For fluorescence observation, cells were exposed for 60 min to alkaline pH (by addition of 100 mM Na_2_HPO_4_ to WMM). The images depicted in Fig. 6A show that, under alkaline conditions, EnaA-GFP displayed preferential plasma membrane localization (PM). A regular distribution of fluorescence was observed at PM of apical and distal hyphal compartments. Thus, response to alkaline pH seems to involve transport to, and regular distribution of EnaA ATPase to all parts of a hypha avoiding a preferential accumulation at the cell tip, where major exocytic and endocytic processes have been shown to occur^31–33^. Fluorescence of EnaA was present at the apical region (t), at branching points (b) and also between compartments, where septa were formed (s), as well as in spots dispersed within the cytosol. At earlier times of induction (15–30 min exposure to pH 8, images at the bottom of panel A), EnaA-GFP fluorescence was visible along the apical compartment but location at PM was not uniform, indicating the existence of clusters of transporters probably associated to specific regions at the PM such as lipid rafts (indicated with white arrowheads and dotted lines).

After induction of the expression of the EnaB-GFP chimera by alkaline pH, microscopy showed a preferential location of EnaB in intracellular, elongated and round structures (Fig. 6B). Elongated structures accumulated at the tip of the cell while round ones distributed at basal locations of hyphal compartment. Noticeably, EnaB-GFP localizations resemble those illuminated by Sec63-GFP, a protein associated to ER and nuclear periphery (possible nuclei, black arrowheads, Fig. 6C^34^). Presence of EnaB at the periphery of nuclei is shown in Supplementary Fig. 3. Thus, EnaA and EnaB have distinct locations, being EnaA located at the PM and EnaB probably in ER as well as additional intracellular membranous compartments such as nuclei, Golgi and transport vesicles.

### Alkaline pH sensitivity of loss of function mutants in the pH regulatory system is not a result of reduced expression of *enaA* gene

The strong dependency of *enaA* expression on the Pal/PacC pathway and its preferential PM localization, led us to speculate that EnaA might be one of the major factors in determining alkaline pH tolerance in the absence of a functional Pal/PacC pathway. Thus, we analyzed whether a PacC independent expression of *enaA* could rescue the alkaline-sensitive phenotype caused by a loss-of-function mutation in the Pal signalling pathway such as *palA1*.

**Figure 7:**
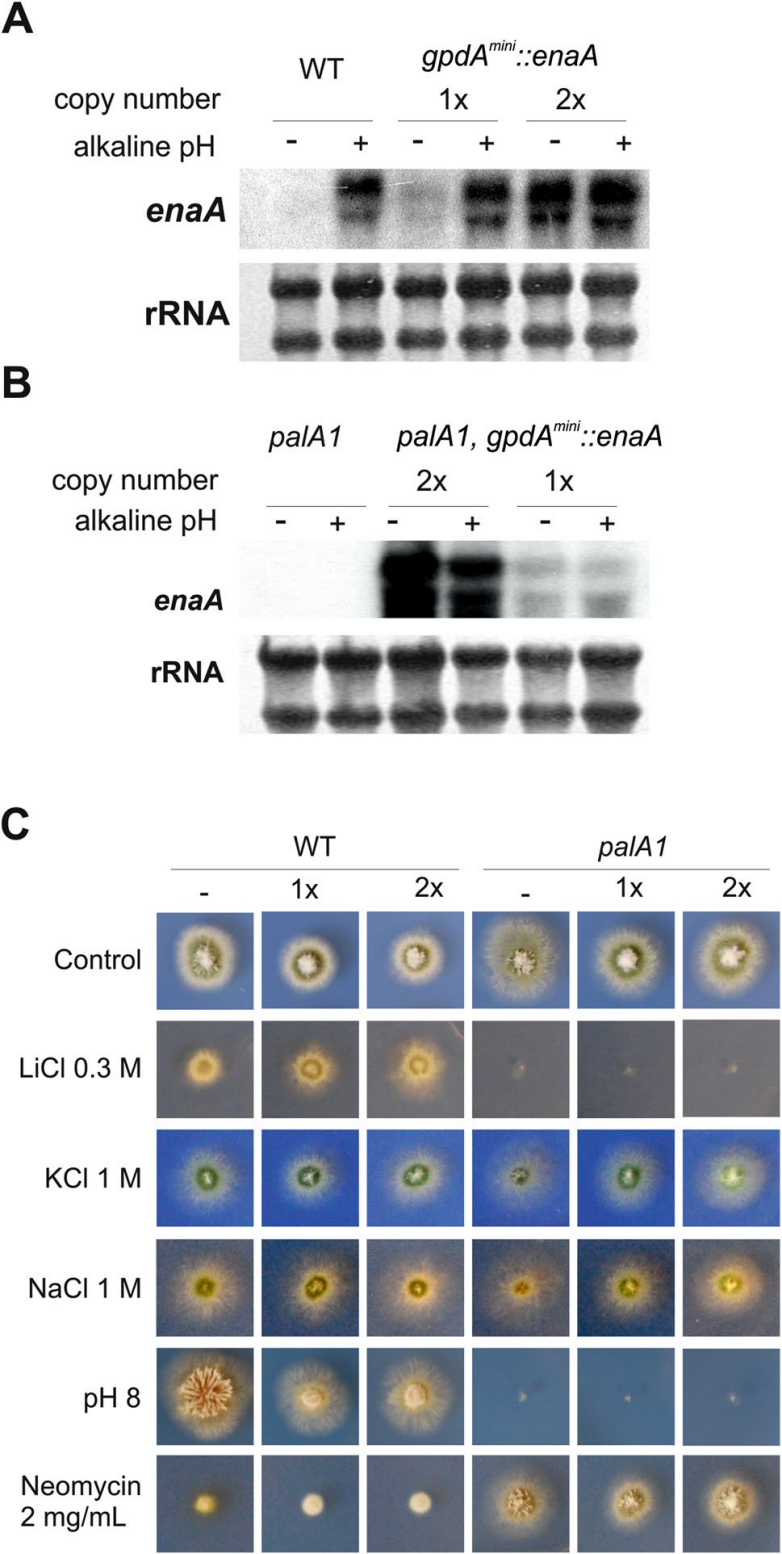
Constitutive expression of *enaA* under the control of the *gpdA*^*mini*^ promoter. (**A**) Expression, in a wild-type background and under inducing (+) or non-inducing (−) conditions, of *enaA* driven by the native or the *gpdA*^*mini*^ promoters. Strains that integrated one or two copies of the *gpdA*^*mini*^*::enaA* plasmid were analyzed. Ribosomal RNA is shown as a loading control. (**B**) Same experiment as in (**A**) but carried out with samples corresponding to the *palA1* mutant background. (**C**) Phenotypes of wild-type (left block of images) or *palA1* (right) strains bearing zero (–), one (1 ×) or two (2 ×) copies of the *gpdA*^*mini*^*::enaA* plasmid after 48 h of culture at 37 °C on AMM (row 1), AMM with LiCl (0.3 M; row 2), KCl (1 M; row 3), NaCl (1 M; row 4) or neomycin (2 mg/mL; row 6), and AMM adjusted to pH 8 (row 5).

To express *enaA* in a pH-independent manner, we transformed a wild-type strain with a plasmid carrying an insert in which the coding region of *enaA* was under the control of a truncated version of the *gpdA* promoter^35^, *gpdA*^*mini*^*,* which leads to constitutive but moderate expression levels^32^. Of selected transformants, MAD3042 carried a single copy and MAD3043 two copies of the construct integrated at the *pyroA locus*. Both strains displayed a wild-type phenotype for salt or alkaline pH tolerance, although, as expected, different expression levels of *enaA* independent of pH condition were detected (Fig. 7A). The amount of *enaA* mRNA increased consistently with the copy number and in the single copy *gpdA*^*mini*^*::enaA* strain *enaA* mRNA levels were similar to those produced by the endogenous *enaA locus* at alkaline pH. Subsequently, both strains were independently crossed with a *palA1* mutant strain (MAD1865)^36^. Progeny carrying one or two copies of *gpdA*^*mini*^*::enaA* and the mutation *palA1* were selected and analyzed (strains I25 (single copy) and I4 (double copy)). Using Northern analysis, we confirmed a constitutive expression of *enaA* independent of inducing conditions and of a functional Pal/PacC pathway in both strains (Fig. 7B). However, phenotypic analysis could not detect any effect of expressing *enaA* in a *palA1* mutant background regarding salt or pH sensitivity (Fig. 7C). Indeed, addition of neomycin (2 mg/mL) to the culture medium showed hyper-tolerance of *palA1* strains expressing *gpdA*^*mini*^-driven *enaA* to this compound, as has been traditionally described for this group of mutants^37^. Therefore, PacC-independent expression of *enaA* does not suppress the sensitivity to alkaline pH, supporting the broad regulatory activity of a functional Pal/PacC pathway and the complexity of alkaline pH response by *A. nidulans*.

## Discussion

In this work we investigated, through a combination of genetics and cell biology techniques, the roles of the three putative sodium ATPases of the ENA family identified in *A. nidulans*^16,19,38^. Sequence similarities, domain distribution and the phylogenetic association (Fig. 1) indicate that *A. nidulans* Ena-like ATPases probably fulfil the same biological function described for several filamentous fungal and yeast ENA homologues. The action of Ena P-type ATPases in fungi has been commonly related to potassium, lithium and sodium ion export from the cytoplasm, especially at alkaline and high salinity conditions^39–41^. However, phylogenic comparison supports the existence of genetic variability of Ena P-type ATPases, which are believed to have evolved from an ancestral K^+^ ATPase by gene duplication in adaptation to salt and/or alkaline conditions^13^. Thus, it is not surprising that evolved functional variants cannot be directly inferred by sequence similarity analyses. This is exemplified by unsuccessful attempts to suppress growth defects in *S. cerevisiae* ENA mutants by expression of an Ena P-type ATPase from *Neurospora crassa*^12^ or from *Hortaea werneckii*^28^. Of the three Ena proteins found in *A. nidulans*, EnaA and EnaB share higher sequence conservation than with EnaC, suggesting a different phylogenetic origin or divergent evolutionary paths.

In contrast to previously cited ENA functions in other organisms, phenotypic analyses of *A. nidulans* null *enaA* strains clearly established that a functional EnaA ATPase is not essential for Na^+^ or for Li^+^ detoxification, but it is necessary at alkaline pH (Fig. 2). A different scenario was found when studying EnaB function. EnaB is necessary for growth in high salinity (Li^+^ and Na^+^) and alkalinity environments. Strong sensitivity to alkalinity is observed in the absence of both EnaA and EnaB proteins, suggesting the existence of overlapping functions of these ATPases in adaptation to alkalinity. At alkaline conditions the importance of EnaB in Na^+^ and Li^+^ tolerance is more prominent, being indispensable in the latter. Thus, the described properties of EnaB are more similar to the function that has been observed for typical Ena-like P-type ATPases. The function of EnaC remains, as yet, undefined. In our phylogenetic analyses, EnaC clustered together with HwENA1 and HwENA2 of *H. werneckii*; however, a requirement of EnaC function in adaptation to alkaline condition or salinity was not observed in this work. This suggests that EnaC undergoes specific spatial and temporal regulations, is activated by other cations or may even have become a pseudogene during Ena evolution in *A. nidulans*.

The above-described differential expression patterns of *A. nidulans* ENA proteins correlate with what has been observed in other fungal species. Indeed, *ena* expression patterns in response to high external ion concentrations and pH differ significantly among orthologues and paralogues of different fungal species. In salt tolerant species such as the Dothideomycete *H. werneckii* (HwENA1 and HwENA2) or the Saccharomycete *Zygosaccharomyces rouxii* (ZENA1), expression of Ena P-type ATPases is induced only at elevated sodium ion concentrations, (5 M and 3 M, respectively^28,41^). In *Debaryomyces* (formerly *Schwanniomyces*) *occidentalis*, ENA1 and ENA2 transcript levels are very low at neutral pH and 0.2 M NaCl concentration^42^. On the other hand, in *S. cerevisiae*, which is more salt sensitive, ENA1 expression is strongly induced by addition to the medium of 0.4 M Na^+^, 0.05–0.1 M Li^+^, as well as by alkaline pH^5^. UmEna1 of *Ustilago maydis* has been characterized as a typical Na^+^ and K^+^ efflux ATPase and is strongly induced by 0.5 M NaCl, whereas UmENA2 is only marginally induced^40^.

The present work highlighted that *A. nidulans ena* genes are differentially regulated by Na^+^, Li^+^ and alkaline pH. We determined that *enaA* is weakly expressed in standard culture conditions, AMM with pH close to neutral (~ 6.8), and is induced by 1.0 M NaCl, 0.3 M Li^+^ and by alkaline pH (8.0) (Fig. 3A). Furthermore, at alkaline pH, EnaA expression is strongly elevated. A similar effect has also been described for UmEna1 and UmEna2 and HwENA1 and HwENA2^28,40^. The highest expression levels for this group of Ena P-type ATPases are achieved when alkalinity is combined with high-Na^+^. However, these high gene expression levels do not correlate with an essentiality of the gene function, as observed in the phenotypic analysis of the null *enaA* strain. *enaB* followed a comparable expression profile to that of *enaA*, although in general with lower concentrations than those of *enaA* (except for basal conditions). Still, differences were observed, especially regarding to Na^+^-induction time, which was later in the case of *enaB*. A hypothetic coordination of an early response mediated by *enaA* and a late response mediated by *enaB* would require both ATPases to efflux Na^+^ at alkaline pH. It is interesting that, despite the essentiality of EnaB on Li^+^ tolerance at neutral and at high pH conditions (Fig. 2), Li^+^ did not induce such a remarkable increase in *enaB* expression as did Na^+^ or alkaline pH.

Our work also focused on the transcriptional regulation of Ena-like ATPases. In *S. cerevisiae,* in addition to the Pal/PacC (Rim101) pathway, the calcineurin/CRZ1 and high osmolarity glycerol (HOG) osmo-responsive pathway are involved in the control of *ENA1* expression together with a regulation by nutrients via Snf1 and Mig1,2 (reviewed in^5,43^). Our previous work demonstrated that *enaA* expression is independent of CrzA^16^. AtfA of *A. nidulans* was characterized as a candidate transcription factor acting downstream of HogA/SakA MAPK and playing a role in oxidative and osmotic stress responses^19,38,44^. Expression of *enaA* has been shown to be independent of AtfA, but *enaB* and *enaC* displayed AtfA-dependent expression especially in response to oxidative stress^38^. This observation establishes a putative link between EnaC and the response to oxidative stress that should be assessed more deeply in the future.

Elevated transcript levels of *enaA* and *enaB* at alkaline pH, together with the presence of several putative binding sites for PacC (5′-GCCAAG-3′) at their promoters, supported a role of the Pal/PacC regulatory pathway and we showed that PacC is an essential positive regulator of *enaA* and *enaB* expression. In the absence of the Pal signalling pathway (*palH72*) that finally activates the PacC transcription factor, neither *enaA* nor *enaB* transcripts were detected. In the constitutive PacC background (*pacC*^*c*^), different transcript levels were obtained to those observed under inducing conditions in the wild type background. This result contrasts with previous reports on the mechanisms PacC-dependent gene regulation (e.g. *ipnA*^45^). The absence of a full upregulated expression of *enaA* and *enaB* in the *pacC*^*c*^*14* background reveals the need of at least one additional regulatory pathway to influence *enaA* and *enaB* expression in response to salinity. A minor positive regulatory role for the salt activated transcription factor SltA on expression of *ena* genes has been shown in this and previous works^16^. This is in agreement with our RNA-seq results^10^, in which both *enaA* and *enaB* (but not *enaC*) were among the most intensely upregulated genes at pH 8 in a wild-type background (Top45 and Top2 genes with the highest log2FC values, respectively), but none of them was significantly downregulated in the *sltA*Δ mutant under these stress conditions.

One possible factor causing the different cation and pH sensitivity of *enaA* and *enaB* null strains could be the different subcellular localization of the proteins. EnaA, as *S. cerevisiae* Ena1p^46^, localizes in the plasma membrane, whereas EnaB localizes in an intracellular membranes system that resembles the ER and Golgi network. However, since Li^+^ is very toxic at low concentrations, compartmentalization into it by EnaB could greatly increase Li^+^ tolerance. Results described in previous reports demonstrated that *Ustilago maydis* UmEna2p and *Neurospora crassa* NcEna2^15,40^ localize in the proximity of the ER and other endomembranes.

We further considered that, since a functional *enaA* or *enaB* was necessary for alkaline tolerance, constitutive expression of *enaA* could be sufficient to rescue the alkaline sensitive phenotype of a Pal/PacC loss-of-function mutation. A strain constitutively expressing *enaA* in a *palA1* genetic background maintained the *palA1* phenotype, however, confirming that other Pal/PacC-controlled elements are required*.* This is in agreement with the envisaged role of PacC as a regulator of multiple pathways in response to environmental pH changes. In this context, next-generation sequencing and analysis tools should help unveil further details of this complex process.

## Methods

### Fungal strains and growth conditions

*Aspergillus* minimal media (AMM) adjusted to pH 6.8, containing 2% glucose and 71 mM sodium nitrate as main carbon and nitrogen sources, respectively, was prepared as described previously^47^. *A. nidulans* strains used in this study are listed in Supplementary Table 1. Strain MAD1427 was used for the systematic deletions. Meiotic crosses and analysis of the progeny followed standard procedures as described previously^48^. Strain BD575 was obtained from the progeny of a cross between MAD2446 and BD488, and strains BD604 and BD612 were obtained from the crossing between BD486 and BD575. Progeny was verified by PCR analysis using oligonucleotides listed in Supplementary Table 2.

Phenotypic analyses of mutant strains were done on solid AMM supplemented with a range of compounds to generate cation or pH stress. Sodium and lithium stress were induced by addition of 1.0 M NaCl and 0.3 M LiCl, respectively. Alkaline stress was induced by adjusting media pH to 8 with either 50 mM Tris–HCl pH 8 or 100 mM Na_2_HPO_4_, always with similar results. Specifically, the use of 100 mM Na_2_HPO_4_ on solid medium was avoided to prevent precipitation when combining with lithium chloride. Radial extension of mutant strains was always compared to that of the reference wild-type strain. Strains were incubated at 37 °C for 2 days and at least 3 replicates per strain and condition were analyzed.

For investigating the effects of alkali-cations and alkaline pH on biomass production, 1 × 10^6^ spores of the mutant strains of interest were inoculated in liquid medium (30 mL of medium in 100 mL flask) with or without addition of the stress agent (1.0 M NaCl, 0.3 M LiCl or 50 mM Tris–HCl pH 8). After cultures were incubated for 24 h at 37 °C in an orbital incubator at 200 rpm, mycelia were collected, dried at 100 °C and weighed. Three biological replicates were performed per strain and condition. For data normalization, growth of each strain at liquid AMM without addition of the stress agent was designated as 100%. Data were presented as percentages. Statistical significance of differences observed in biomass production between the wild-type and mutant strains was evaluated using Two-tailed Student’s *t*-test for unpaired samples.

### Construction of deletion cassettes for *enaA*, *enaB* and *enaC*

Gene deletion was achieved by using deletion cassettes comprising *Aspergillus fumigatus pyrG *(AN6642/*enaA;* AN10982*/enaC*),* riboB *(AN1628/*enaB*), or *pyroA* (AN10982/*enaC*) genes, respectively*,* as selectable markers flanked by 700–1,500 bp up- and downstream of the targeted *A. nidulans* open reading frame. The selectable markers were amplified with primer pairs gsp2*-gsp3* (listed in Supplementary Table 2), which contained tails (20 bp) specific to each terminal of both the 5′ and 3′ ends of the targeted gene, and using the cloned selectable marker as a template present on plasmids (see Supplementary Table 3). Fragments corresponding to the 5′-UTR and 3′-UTR of the targeted genes were amplified using genomic DNA of *A. nidulans* as template and oligonucleotide pairs gsp1–gsp2 or gsp3–gsp4, respectively. The final cassette was generated by fusing the three fragments by Fusion PCR using primers gsp1–gsp4^49^. Cassettes were purified and directly used to transform *A. nidulans* protoplasts (Supplementary Table 1) following the protocol described previously^50^. The double-null mutants were generated by sequential transformations of strains deficient in heterologous recombination^51^ with the required fusion cassettes. Prototrophic transformants were isolated and deletion of the targeted open reading frame confirmed by Southern-blot^52^ after genomic DNA extraction^53^.

### GFP tagging of EnaA and EnaB

Construction of *A. nidulans* strains expressing C-terminally GFP-tagged proteins followed the 3-way PCR methodology described previously^49^, ~ 1.5 kb from the most 3′ end of the target gene and the 3′ UTR region were amplified with primers gsp5–gsp6 and gsp3–gsp4, respectively (Supplementary Table 2), and fused in a 3-way PCR with a fragment, amplified with primers gsp6*–gsp3*, containing the *gfp* gene together with the selectable *pyrG* gene of *A. fumigatus*^49^. The fusion cassette was transformed into recipient MAD1427 strain that carries *pyrG89* mutation. Uracil and uridine prototrophs were selected, purified to homokaryosis and then analyzed by Southern-blot for verification of a single integration event of the gene replacement cassette. Functional replacement was verified by phenotypic analysis using wild-type and deletion phenotypes as references.

### Constitutive expression of *enaA*

The *enaA* coding sequence was amplified using primers EnaAup and EnaAdown, which introduced *Eco*RI restrictions sites for further cloning into plasmid p1660^32^. This plasmid carries a truncated version of *gpdA* promoter, *gpdA*^*mini*^, allowing constitutive expression of target gene, and a truncated version of *pyroA* gene, restricting integration at the *pyroA* locus by complementation of *pyroA4* mutation (Table Supplementary 3). Selection of positive transformants is based on recovery of pyrimidine prototrophy (see also^54^). The resulting plasmid was named pGPDA-EnaA and the integrity of the *enaA* open reading frame was confirmed by sequencing. Protoplasts of strain MAD1739 were transformed using pGPDA-EnaA and positive transformants and copy number of integration events at the *pyroA* locus were confirmed by Southern blot analysis.

### RNA extraction and gene expression analyses

For RNA extraction, 1 × 10^6^ spores of wild-type and single null mutant strains were inoculated in fermenters and cultured for 16 h at 37 °C and 200 rpm. Mycelia were harvested and transferred to flasks with fresh medium containing 1.0 M NaCl, 0.3 M LiCl or pH 8.1, with further incubation of the culture as specified in the figures. Mycelia samples were collected by filtration, frozen in liquid nitrogen and grounded.

Total RNA was isolated following manufacturer’s protocol by adding 1 mL TRIzol reagent (Fluka, Sigma-Aldrich Quimica SL) to 100 mg of grounded samples. Total RNA concentration was calculated using a Nanodrop 2000c system (Thermo Fisher Scientific, Waltham, MA). Northern blot assays were carried out as described previously^55^. 10 μg of total RNA per sample was loaded in 1.2% agarose gels and transferred to positively charged nylon filters (Roche). Equal loading of total RNA was evaluated by methylene blue staining of rRNA. Transcripts of *A. nidulans ena-*like genes were detected using specific PCR-amplified probes as described previously^16^. mRNA levels of *msnA* gene (AN1652) were detected using a probe of 1,995 bp amplified using primers described in Table S2 and which covered 100% of the ORF. Labelling of DNA probes was performed using Roche radioactive labelling kit, detected using a PhosphorImager screen (Molecular Dynamics) and developed using a FLA-5100 Reader (Fujifilm). Band intensity quantification was performed using Multi-Gauge V3.0 software (Fujifilm).

### Protein isolation and western blot

Sodium, lithium and alkalinity-shock effects on protein concentration was studied using western-blot technique. Total protein extracts were isolated from mycelium of GFP-tagged strains cultivated for 16 h at 37 °C in appropriately supplemented Cove’s minimal medium^56^ plus the indicated time after addition of the stress agent. Mycelia were harvested by filtration, frozen in dry ice and lyophilized for 16 h. Protein extraction from lyophilized samples was performed by alkaline-lysis extraction procedure described previously^22^. Proteins were resolved in 8% SDS–polyacrylamide gels and transferred to nitrocellulose membrane using TransBlot^®^ Turbo™ System (Bio-Rad). GFP-tagged versions of EnaA and EnaB were detected using mouse anti-GFP monoclonal antibody (1:5,000 dilution; Roche). As loading control, gamma-actin was detected using mouse anti-actin C4 antibody (1:5,000 dilution; MP Biomedicals). As secondary antibody peroxidase-conjugated goat anti-mouse IgG immunoglobulin (1:4,000 dilution; Jackson) was used. Western blots were developed using ECL kit (GE Heathcare) and images were taken using a Luminescent Image Analyzer LAS-3000 (Fujifilm) and processed with Multi-Gauge V3.0 software (Fujifilm).

### Fluorescence microscopy

*Aspergillus nidulans* conidiospores were inoculated in uncoated glass-bottom µ-dishes (Ibidi GmbH) containing 2.5 mL watch minimal medium (WMM^57^) supplemented with 25 mM NaH_2_PO_4_, 5 mM ammonium (+)-tartrate, and 0.5% glucose. After 16 h at 25 °C medium was replaced with freshly prepared WMM supplemented with 100 mM Na_2_HPO_4._ Samples were cultured for an additional 1.5 h at 37 °C prior to observation of fluorescence (see text for details of *enaA* and *enaB* induction).

Differential Interference Contrast (Normarski optics) and fluorescence images were acquired from in vivo cultures with a Leica DMI-6000b inverted microscope coupled to an ORCA-ER digital camera (Hamamatsu Photonics) and equipped with a 63 Plan Apochromat 1.4 N.A. oil immersion objective (Leica) and a GFP filter (excitation 470 nm; emission 525 nm). Images were acquired using Metamorph (Molecular Dynamics) software and processed using ImageJ free-software (https://imagej.nih.gov/ij).

### Bioinformatics

Multiple sequence alignments were made using BLAST and Clustal Omega open source program (https://www.ebi.ac.uk/Tools/msa/clustalomega/). Jalview application was used for alignment visualization and domain analyses. Transmembrane domains of Ena-like proteins were predicted using Hidden Markov Models (HMM) in the Institute Pasteur Mobyle server (https://mobyle.pasteur.fr/). Phylogenetic trees were generated using Mega Version 7.0 software^58^. Maximum-Likelihood (80 replicates) and Neighbor-Joining (10,000 replicates) methods rendered the same organization of the clades. The Maximum-likelihood tree was edited using iTOL^59^.

## Supplementary information

Supplementary Information.
