# Supplementary material for: Tolerance to alkaline ambient pH in Aspergillus nidulans depends on the activity of ENA proteins
Source: Sci Rep. 2020 Aug 31;10:14325. doi: 10.1038/s41598-020-71297-z (PMC7459330; doi:10.1038/s41598-020-71297-z)
Supplement: Supplementary file 1 — Supplementary Information. [file 41598_2020_71297_MOESM1_ESM.pdf]

Supplementary Information

Tolerance to alkaline ambient pH in *Aspergillus nidulans* depends on the activity of ENA proteins.

Ane Markina-Iñarrairaegui[1], Anja Spielvogel[2], Oier Etxebeste[1] (0000-0002-9786-6091), Unai Ugalde[1] and Eduardo A. Espeso[2,*] (0000-0002-5873-6059)

1. Department of Applied Chemistry, Faculty of Chemistry, University of the Basque Country, San Sebastian, Spain

2. Department of Cellular and Molecular Medicine, Centro de Investigaciones Biológicas Margarita Salas, C.S.I.C., Ramiro de Maeztu, 9, Madrid 28040, Spain

* Correspondence to: eespeso@cib.csic.es.

Running title: Role of P-type ATPases in alkaline pH response.

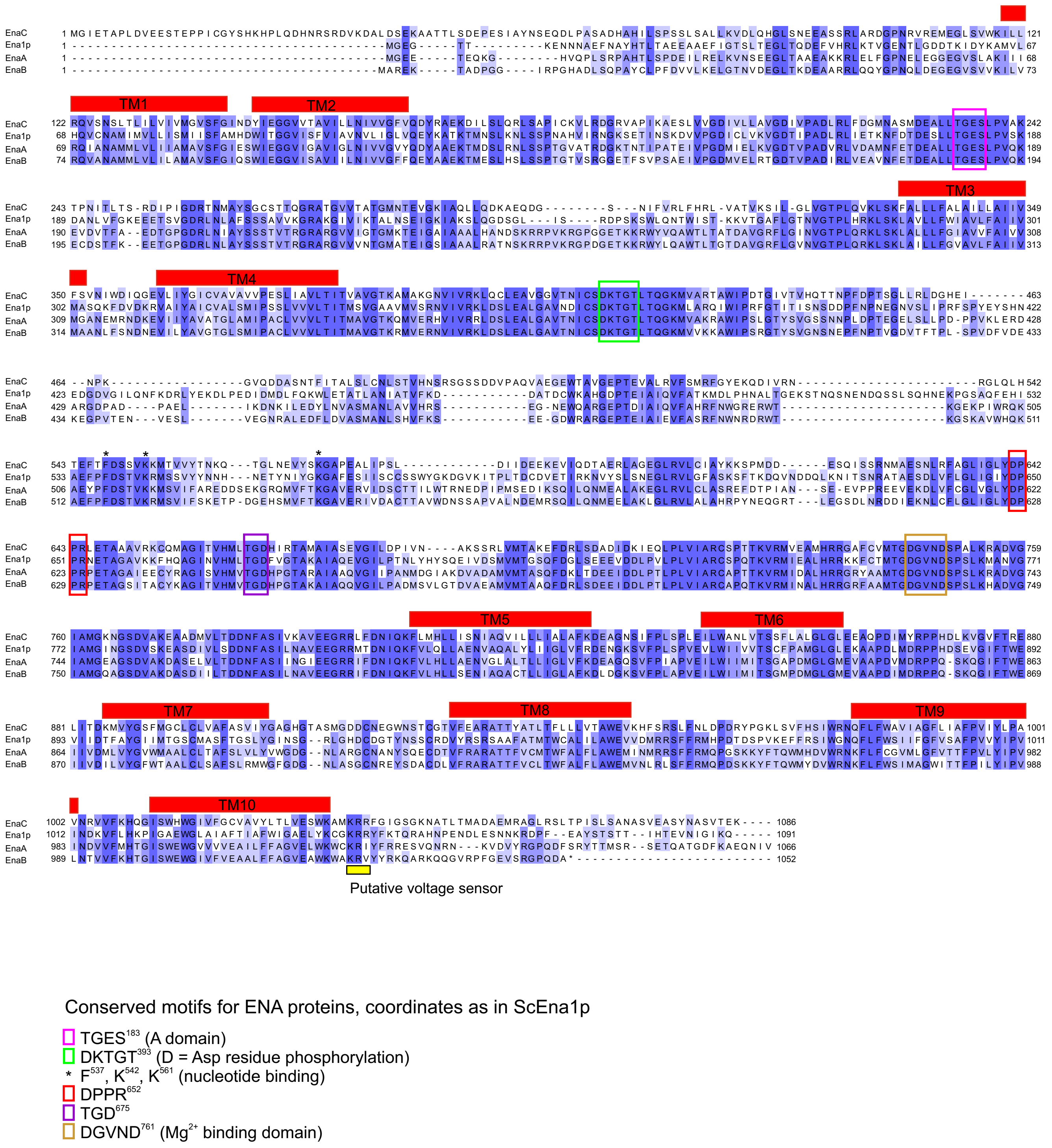

Supplementary Figure 1. Amino acid sequence analysis of An-ENA like ATPases. (**A**) Sequence alignments of *S. cerevisiae* Ena1p and *A. nidulans* predicted full-length ENA ATPases. Clustal method was used for the comparison. Boxes describe conserved functional regions detailed in reference [1] and the red rectangles delimit the position of the putative transmembrane regions predicted with Hidden Markov Models (TMHMM and HMMTOP). Protein accession numbers are reported as follows: *Saccharomyces cerevisiae* Ena1p (NP_010325), *Aspergillus nidulans* EnaA (CBF71157), EnaB (CBF85251) and EnaC (CBF79858).

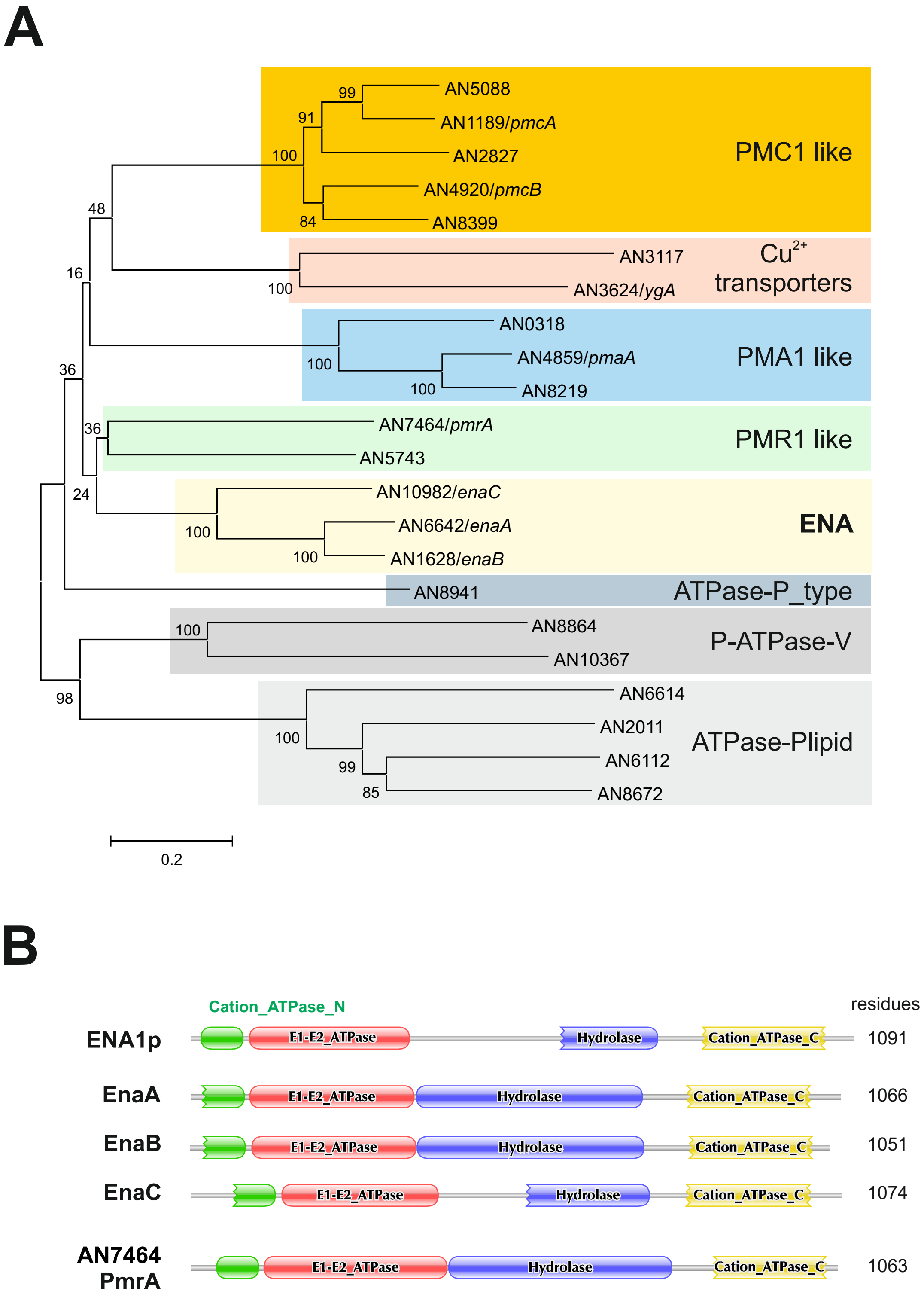

Supplementary Figure 2. Classification of ATPases in *A. nidulans* and distribution of functional domains. (A) Phylogenetic tree corresponding to 22 ATPases of *A. nidulans*. The tree was generated using Mega (version 7.0) [2] and the Neighbor-Joining method, with a bootstrap test value of 10,000 replicates. EnaA, EnaB and EnaC group to the same clade of the tree, next to PMR1-like proteins. (**B**) Representation of domain organization and extension of EnaA, EnaB and EnaC according to Pfam, AspGD and FungiDB databases. Colours keyed to cation-ATPase-N (pfam00690) domain (green), E1-E2_ATPase (pfam00122) domain (red), Hydrolase, haloacid dehalogenase-like hydrolase (HAD) (COG4087. pfam00702) domain (blue), and Cation_ATPase_C (pfam00689) domain (yellow).

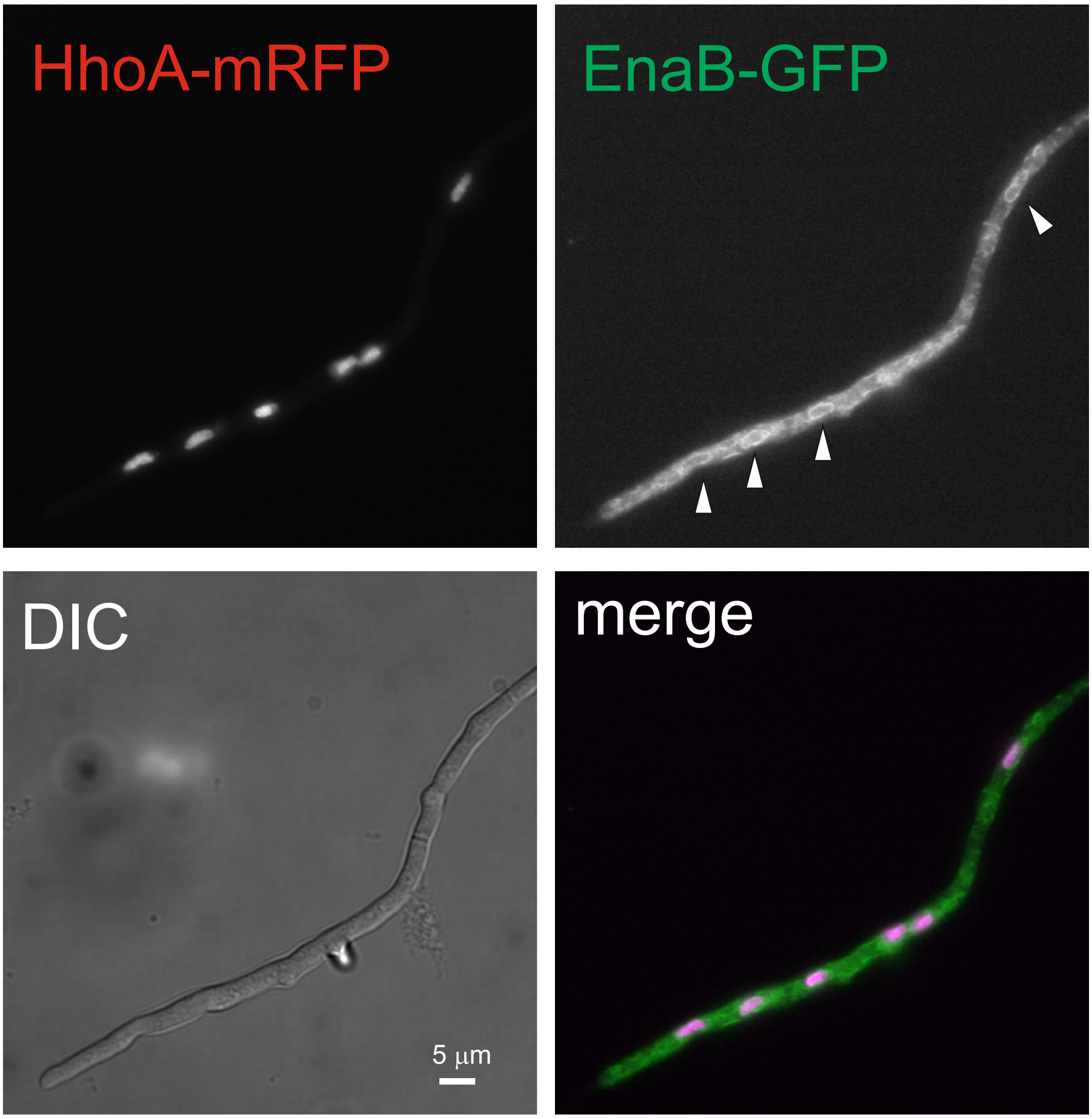

Supplementary Figure 3. Colocalization of EnaB-GFP and nuclei. Images showing the localization of EnaB-GFP and histone H1-mRFP in cells of diploid strain DIP5. Red fluorescence channel is shown on top left image showing a hypha with 6 nuclei. Top right is shown the green fluorescence channel (EnaB-GFP) and the ovoid structures are indicated with white arrowheads. Down-right is the merged image of red and green channels (magenta nuclei/histone H1 and green EnaB) showing the localization of EnaB at the periphery of nuclei. Down left is the Nomarsky image of the hypha and the scale bar representing 5 µm.

Supplementary Table 1. List of *A. nidulans* strains used in this work

Strain	*Genotype*	Source
MAD2446	*pyrG89; wA4; inoB2, pyroA4; hho::mCh::pyroA ;(myc)3-pacC(pacC900); veA1*	Madrid's collection
MAD2173	*pyrG89 argB2 pyroA4Dnku::argB sec63::gfp::pyrGAf,*	[3]
MAD1425	*pyrG89; argB2; pyroA4, nkuAΔ::argB; veA1*	TNO02A3 [4]
MAD1425+ /BD377	*pyrG^{An}; argB2; pyroA4, nkuAΔ::argB; veA1*	[5]
MAD1427	*pyrG89, pabaA1; argB2; nkuAΔ::argB; veA1, riboB2*	TNO02A25 [4]
MAD2732	*pyrG^{An}, pabaA1; argB2; nkuAΔ::argB; veA1, riboB2*	[5]
MAD2731	*pyrG89, pabaA1; argB2; nkuAΔ::argB; veA1, riboB^{An}*	[5]
BD604	*pyrG89, enaAΔ::pyrG^{Af}; argB2; pyroA4, nkuAΔ::argB; veA1*	This work
BD487/BER53-B	*pabaA1; argB2; nkuAΔ::argB; enaBΔ::riboB^{Af}; veA1, riboB2*	This work
BD486/BER55-B	*pyrG89, pabaA1; argB2; enaCΔ::pyrG^{Af}, ΔnkuA::argB; veA1, riboB^{An}*	This work
BD488/BER54	*pyrG89, pabaA1, enaAΔ::pyrG^{Af}; argB2; ΔnkuA::argB; enaBΔ::riboB^{Af} ; veA1, riboB2*	This work
BD489/BER56	*pyrG89, pabaA1; argB2; enaCΔ::pyrG^{Af}, ΔnkuA::argB; enaBΔ::riboB^{Af} ; veA1, riboB2*	This work
BD625	*pyrG89, enaAΔ::pyrG^{Af}; argB2; enaCΔ::pyroA^{Af}, pyroA4, nkuAΔ::argB; veA1*	This work
BD575	*pyrG89, enaAΔ::pyrG^{Af}; pyroA4, nkuAΔ::argB; enaBΔ::riboB^{Af}; inoB2; wA4, argB2*	This work
BD612	*pyrG89, enaAΔ::pyrG^{Af}; argB2; enaCΔ::pyrG^{Af}, pyroA4, nkuAΔ::argB; enaBΔ::riboB^{Af}; veA1*	This work
MAD305	*pabaA1; pacC^{c}14*	Madrid's collection
MAD1074	*inoB2; palH72*	Madrid's collection
MAD4097 /HHF27b	*sltAΔ::riboB^{f}*	[6]
MAD25ED	*pyrG89, enaA::gfp::pyrG^{Af}; pyroA4, nkuA::bar*	This work
MAD26ED	*pyrG89; pyroA4, nkuAΔ::bar; enaB::gfp::pyrG^{Af}*	This work
MAD1739	*pyrG^{An}; pyroA4, nkuAΔ::bar*	Madrid's collection
MAD3042	*pyrG89; pyroA4, nkuAΔ::bar, [pyroA+::gpdA^{mini}::enaA]*	This work
MAD3043	*pyrG89; pyroA4, nkuAΔ::bar, [pyroA+::gpdA^{mini}::enaA]x2*	This work
MAD1865	*pabaA1; pyroA4; inoB2, glrA1, palA1*	Madrid's collection
I25	*pabaA1;[pyroA+::gpdA^{mini}::enaA]; palA1*	This work
I4	*pabaA1; [pyroA+::gpdA^{mini}::enaA]x2; palA1*	This work
DIP5	*pyrG89; pyroA4, nkuAΔ::bar; enaB::gfp::pyrG^{Af} /*	This work

	pabaA1, hhoA::mRFP::pyrG^{Af}	

Supplementary Table 2. Oligonucleotides used in this work

**Name**	**Sequence (5´-3´)**	**Aim**	**Source**
EnaA-gsp1	CGGAAAGACCTTACCATGATTCGC	EnaA 5´ UTR	This work
EnaA-gsp2	CTGACGTTACTACTATGGTGTC	EnaA 5´ UTR	This work
EnaA-gsp3	GCACTCCGACGGAAAGCTTGCTACC	EnaA 3´ UTR	This work
EnaA-gsp4	CAACCACAACCTCGATACTTCGC	EnaA 3´ UTR	This work
EnaA-gsp5	TCTACGATCCGCCTAGGCCTGAGACAGC	EnaA 3´ end ORF	This work
EnaA-gsp6	AACGATATTCTGTTCCGCCTTGAAGTCTCC	EnaA 3´ end ORF	This work
EnaA-gsp2*	GACACCATAGTAGTAACGTCAGACCGGTCGCCTCAAACAATGCTCT	pyrG^{Af} cassette	This work
EnaA-gsp3*	GGTAGCAAGCTTTCCGTCGGAGTGCGTCTGAGAGGAGGCACTGATGCG	pyrG^{Af} /gfp::pyrG^{Af} cassette	This work
EnaA-gsp6*	GGAGACTTCAAGGCGGAACAGAATATCGTTGGAGCTGGTGCAGGCGCTGGAGCC	gfp::pyrG^{Af} cassette	This work
EnaB-gsp1	CAACAACCAGCTCAGCCGTTTGC	EnaB 5´ UTR	This work
EnaB-gsp2	TGACAACTGAGATGATAAGCCCG	EnaB 5´ UTR	This work
EnaB-gsp3	CGTCTGCTTTCCCTCGCTTAG	EnaB 3´ UTR	This work
EnaB-gsp4	AGCTAGCCCTCCAAGCCAACAC	EnaB 3´ UTR	This work
EnaB-gsp5	AACTCATCGGCTCCTGTGGC	EnaB 3´ end ORF	This work
EnaB-gsp6	GGCATCCTGAGGGCCCCTGCTTACCTC	EnaB 3´ end ORF	This work
EnaB-gsp2*	CGGGCTTATCATCTCAGTTGTCAACCGGTCGCCTCAAACAATGCTCT	riboB^{Af} cassette	This work
EnaB-gsp3*	CTAAGCGAGGGAAAGCAGACGGTCTGAGAGGAGGCACTGATGCG	riboB^{Af}/gfp::pyrG^{Af} cassette	This work
EnaB-gsp6*	GAGGTAAGCAGGGGCCCTCAGGATGCCGGAGCTGGTGCAGGCGCTGGAGCC	gfp::pyrG^{Af} cassette	This work
EnaC-gsp1	GGAAAGAGTGCGGGGC	EnaC 5´ UTR (enaC-PP1)	This work
EnaC-gsp2	CATTCAATTGGAGTGGAGGTG	EnaC 5´ UTR (enaC-PP2)	This work
EnaC-gsp3	TAGGGAACGGATGTTACTTCCG	EnaC 3´ UTR	This work
EnaC-gsp4	CGCCGAGATCTCCCG	EnaC 3´ UTR	This work
EnaC-gsp2*	CACCTCCACTCCAATTGAATGCGTGGAGTTACCAGTGATTGACCAGG	riboB^{Af} / pyrG^{Af} cassette (enaC-SMP1)	This work
EnaC-gsp3*	CGGAAGTAACATCCGTTCCCTAGTCTGAGAGGAGGCACTGATGCG	riboB^{Af} / pyrG^{Af} cassette (enaC-SMP2)	This work
RiboAnid term rev	CCTAGCAGTGGTTGAATAGT	*A. nidulans* AN0670.2 Amplification	This work
RiboAnid prom fw	GATAAGTGTTGGTGGAAGTG	*A. nidulans* AN0670.2 Amplification	This work
EnaA-sense	CCTAGGCGTTGAAATCTCCC	Meiotic recombination confirmation	This work
EnaA-antis	GCTCGAAGGTGCGGG	Meiotic recombination confirmation	This work

EnaB-sense	GGCTTGACCTTCGAATCTGC	Meiotic recombination confirmation	This work
EnaB-antis	CTTACCAACAGGCCACACTCC	Meiotic recombination confirmation	This work
EnaC-sense	CGCTCCTCCTGCAATCC	Meiotic recombination confirmation	This work
EnaC-antis	CGAGCGTGTAGGCAGAGG	Meiotic recombination confirmation	This work
An6642 fw	TCAACGTCGGAACACCTCTT	*enaA* forward primer for probe	[7]
An6642 rev	TTGACACCGTCACCAGTCAT A	*enaA* reverse primer for probe	[7]
An1628 fw	TCGTCGTTGCAGGTGCTGAT	*enaB* forward primer for probe	[7]
An1628 rev	TATTGGTCACCGCTCCAAGG	*enaB* reverse primer for probe	[7]
An10982 fw	AGGACATCCTCTCGCTACAG	*enaC* forward primer for probe	[7]
An10982 rev	TCCTTCTCCAGCCAGTCTCT	*enaC* reverse primer for probe	[7]
msnA fw	CGACCATCATCTAATGTGAGTGC	*msnA* forward primer for probe	This work
msnA rev	GAGACCAGTGTGGATGTCGAAGC	*msnA* reverse primer for probe	This work
EnaAup	CGGAATTCATGGGAGAGGAGACCGAAC	*enaA* forward primer with EcoRI site for *gpda::enaA* construction	This work
EnaAdown	CGGAATTCTCAAACGATATTCTGTTCC	*enaA* reverse primer with EcoRI site for *gpda::enaA* construction	This work

Supplementary Table 3. List of plasmids used in this work

Plasmid ID Madrid's collection	Main features	Reference
p1439	Plasmid pFNO3 containing the insert GA$_5$-GFP-*pyrG*^{Af}	[8]
p1547	pBluescriptSK(+) derivative containing *A fumigatus pyroA* gene	Gift from B. Oakley
p1548	pBluescriptSK(+) derivative containing *A fumigatus riboB* gene	Gift from B. Oakley
p1660	Expression vector, harbouring *gpdA* mini promoter, used for moderate expression of target genes.<br>Directed integration to *pyroA4* mutant allele.	[9]

References

1. Palmgren, M.G. & Nissen, P. P-type ATPases. Annu. Rev. Biophys. **40**, 243-266 (2011).

2. Kumar, S., Stecher, G., & Tamura, K. MEGA7: Molecular Evolutionary Genetics Analysis Version 7.0 for Bigger Datasets. Mol Biol. Evol. **33**, 1870-1874 (2016).

3. Markina-Iñarrairaegui, A., Pantazopoulou, A., Espeso, E.A., & Peñalva, M.A. The *Aspergillus nidulans* peripheral ER: disorganization by ER stress and persistence during mitosis. PLoS. One. **8**, e67154 (2013).

4. Nayak, T., *et al.* A versatile and efficient gene-targeting system for *Aspergillus nidulans*. Genetics **172**, 1557-1566 (2006).

5. Markina-Iñarrairaegui, A., *et al.* Nuclear transporters in a multinucleated organism: functional and localization analyses in *Aspergillus nidulans*. Mol. Biol. Cell **22**, 3874-3886 (2011).

6. Findon, H., *et al.* Analysis of a novel calcium auxotrophy in *Aspergillus nidulans*. Fungal. Genet. Biol. **47**, 647-655 (2010).

7. Spielvogel, A., *et al.* Two zinc finger transcription factors, CrzA and SltA, are involved in cation homoeostasis and detoxification in *Aspergillus nidulans*. Biochem. J. **414**, 419-429 (2008).

8. Yang, L., *et al.* Rapid production of gene replacement constructs and generation of a green fluorescent protein-tagged centromeric marker in *Aspergillus nidulans*. Eukaryot. Cell **3**, 1359-1362 (2004).

9. Pantazopoulou, A. & Peñalva, M.A. Organization and dynamics of the *Aspergillus nidulans* Golgi during apical extension and mitosis. Mol. Biol. Cell **20**, 4335-4347 (2009).

Full size images for hybridations of wild type samples filter
See Figure 3 panel A and legend for lanes information.

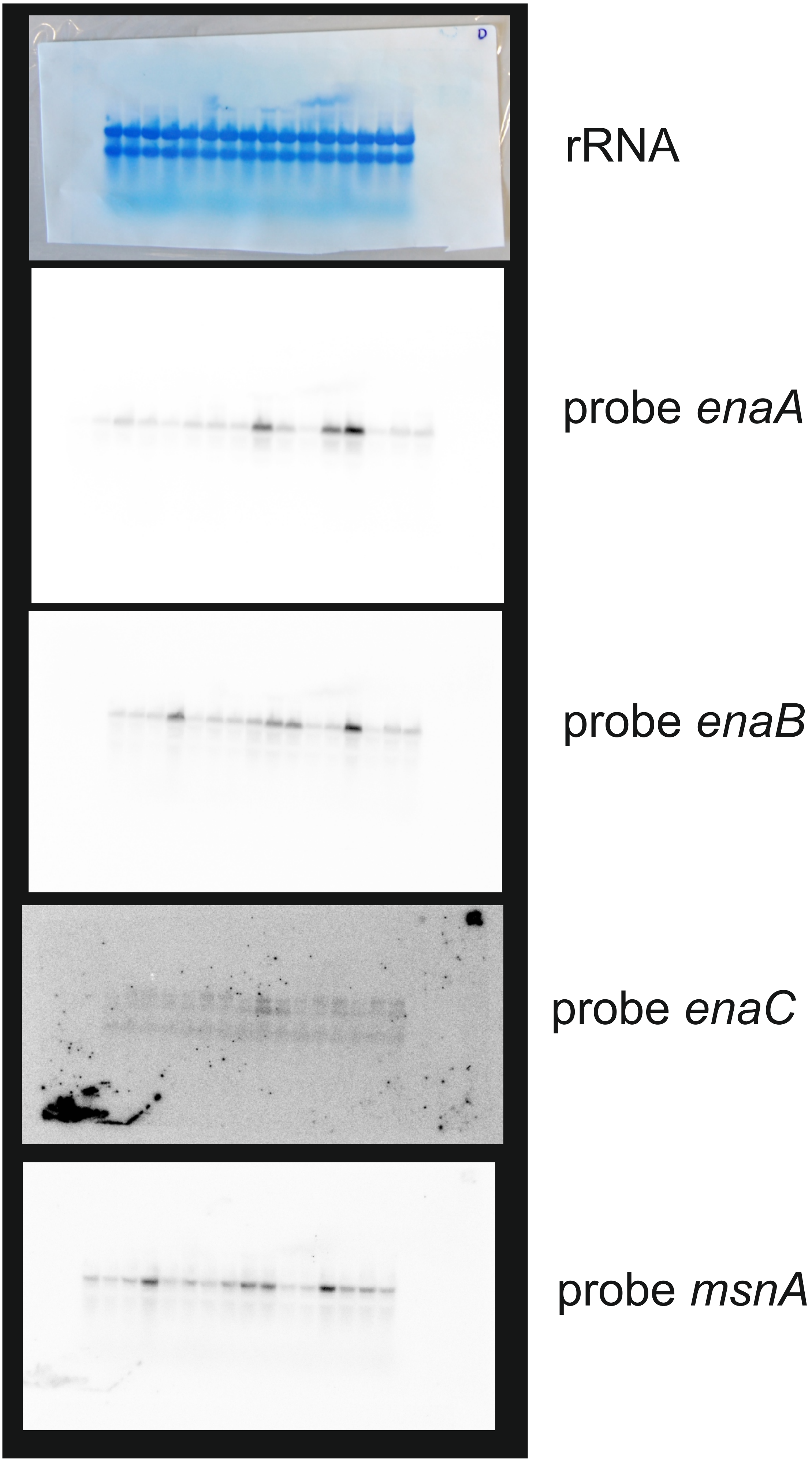

Full size images for hybridations of *enaA*Δ samples filter
See Figure 3 panel C and legend for lanes information.

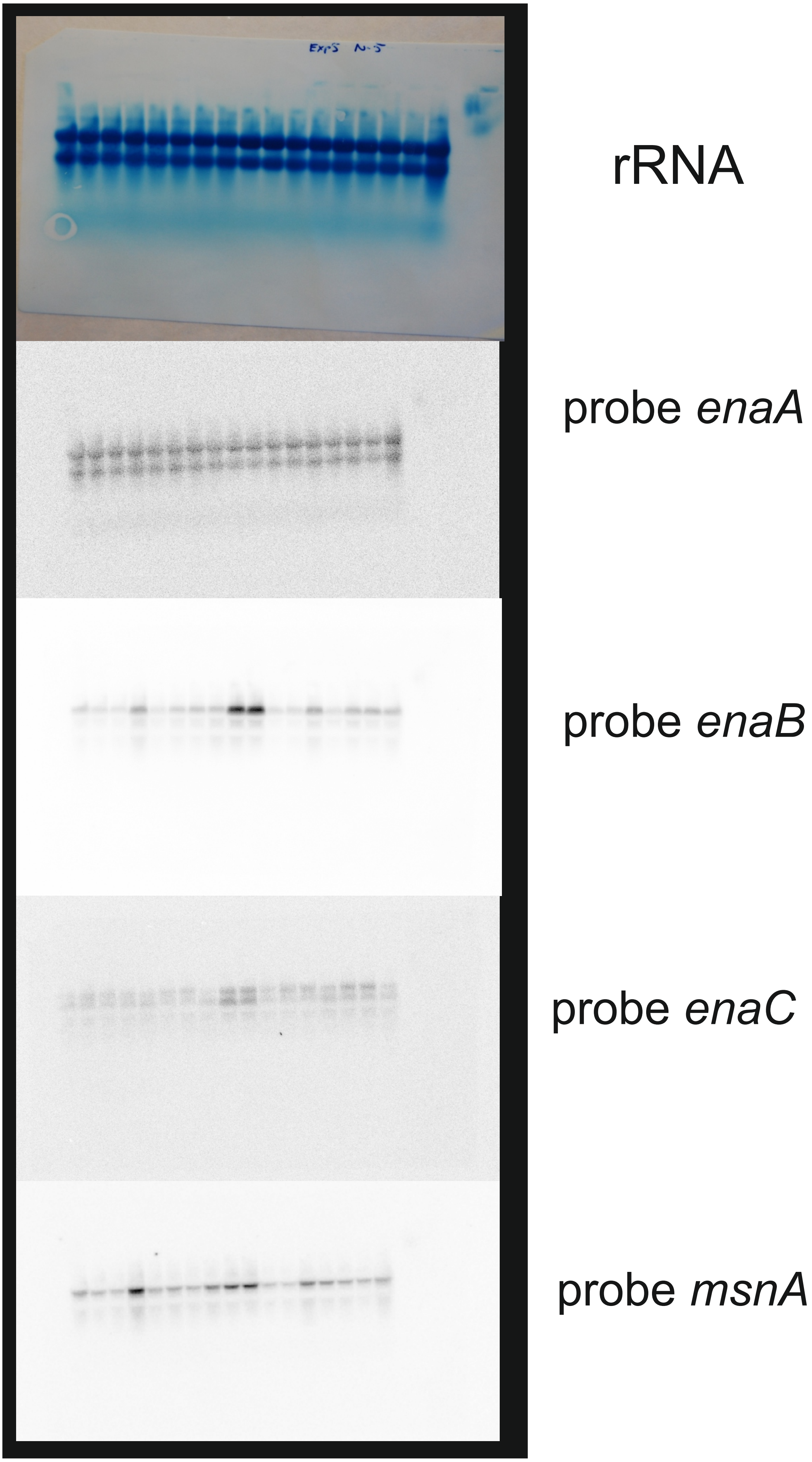

Full size images for hybridations of *enaB*Δ samples filter
See Figure 3 panel D and legend for lanes information.

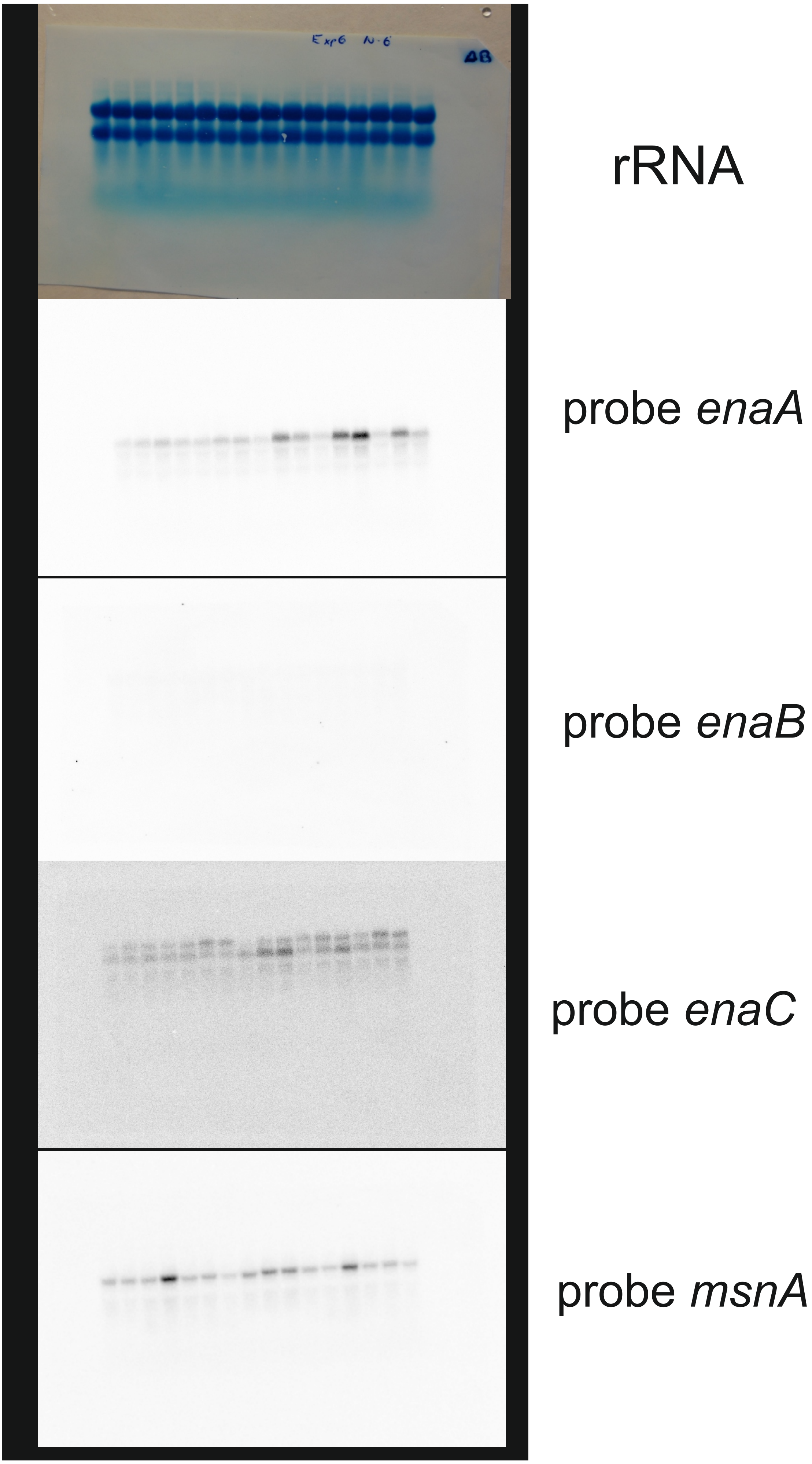

Full size images for hybridations of *palH72* samples filter
See Figure 4 panel B and legend for lanes information.

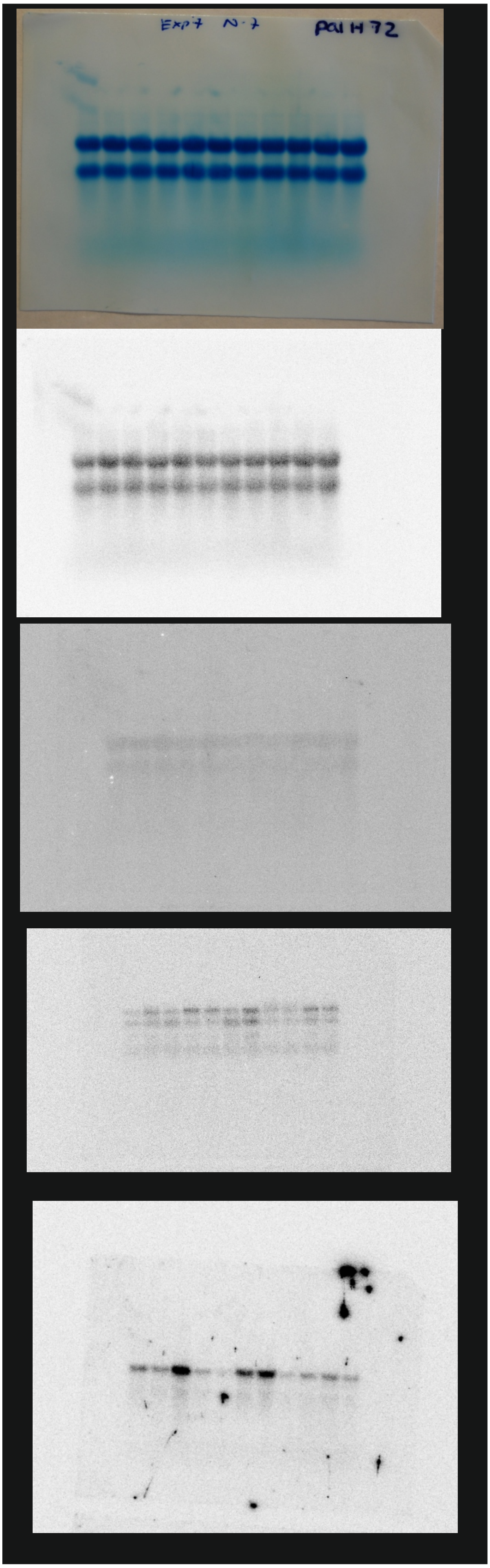

rRNA

probe *enaA*

probe *enaB*

probe *enaC*

probe *msnA*

Full size images for hybridations of *pacC*^{c}*14* samples filter
See Figure 4 panel C and legend for lanes information.

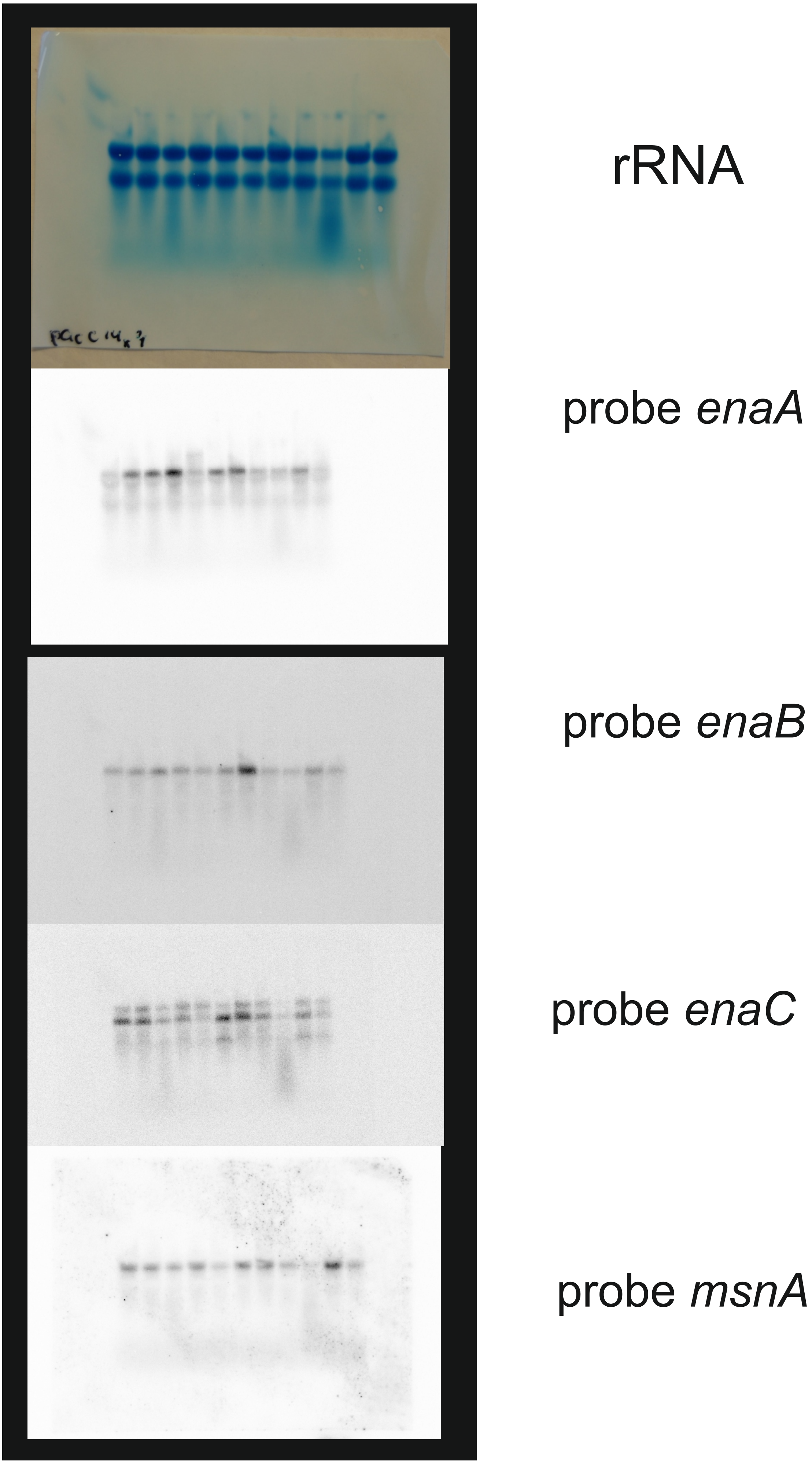

Full size images for hybridations of *sltA*Δ samples filter
See Figure 4 panel D and legend for lanes information.

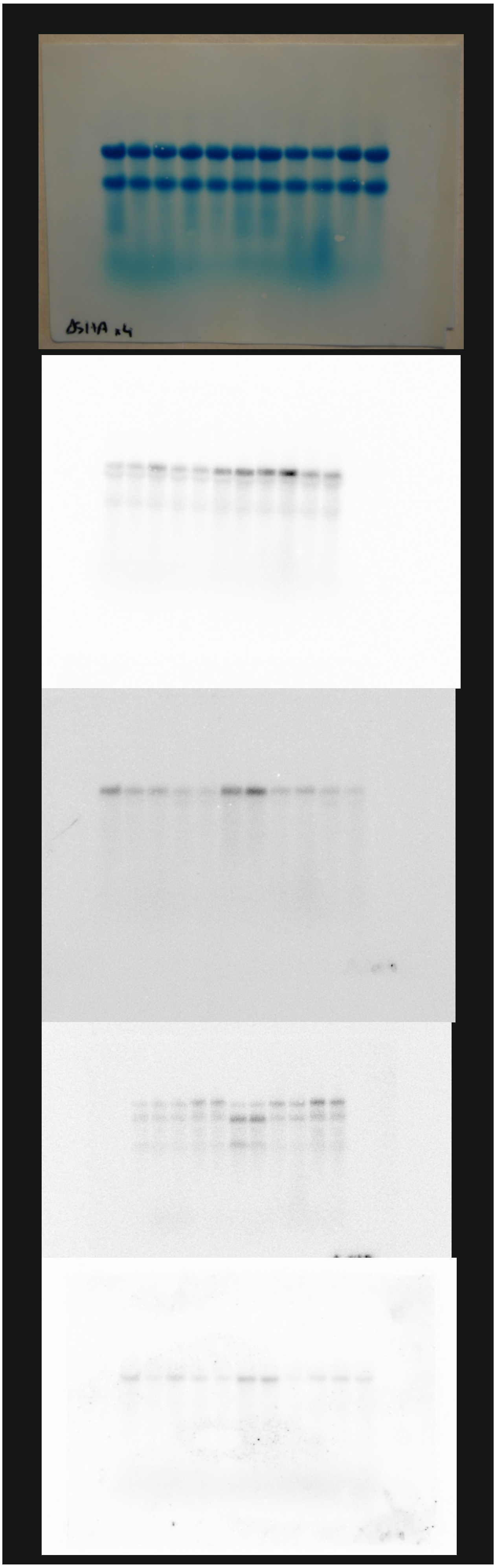

rRNA

probe *enaA*

probe *enaB*

probe *enaC*

probe *msnA*

Full size images for MW markers on filter and immunodetection of GFP and actin in protein extracts from EnaA and EnaB-GFP expressing strains. See Figure 5 panel A and legend for lanes information.

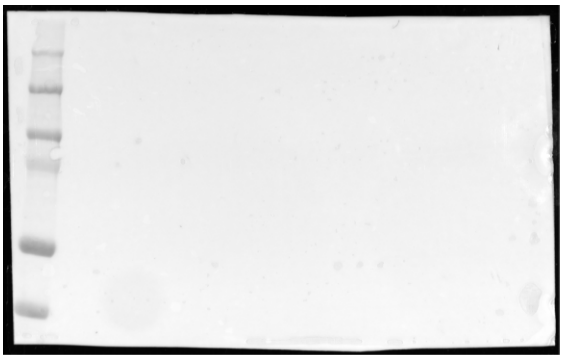

Filter with prestained MWs

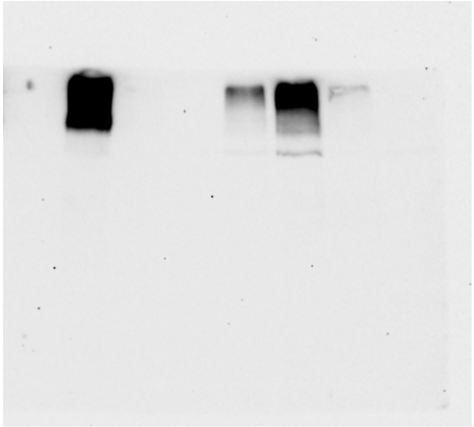

anti-GFP

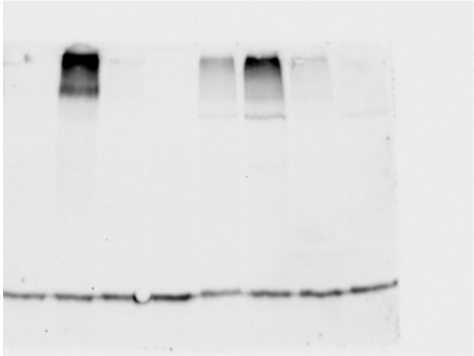

antiGFP + anti-actin

Full size images for MW markers on filter and immunodetection of GFP and actin in protein extracts from EnaA and EnaB-GFP expressing strains. See Figure 5 panel B and legend for lanes information.

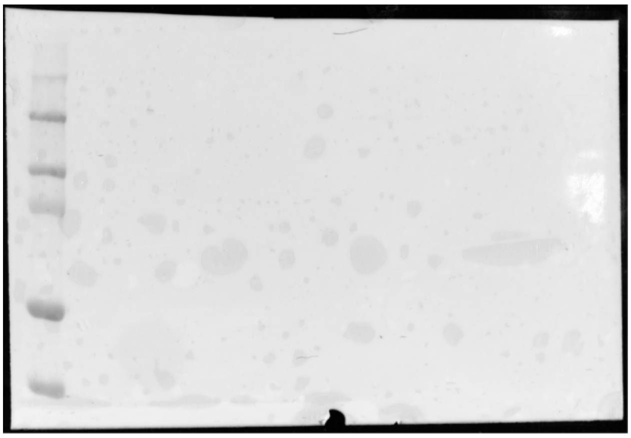

Filter with prestained MWs

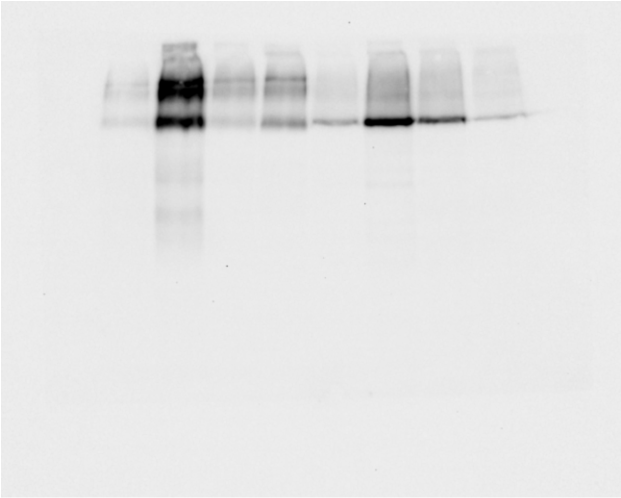

anti-GFP

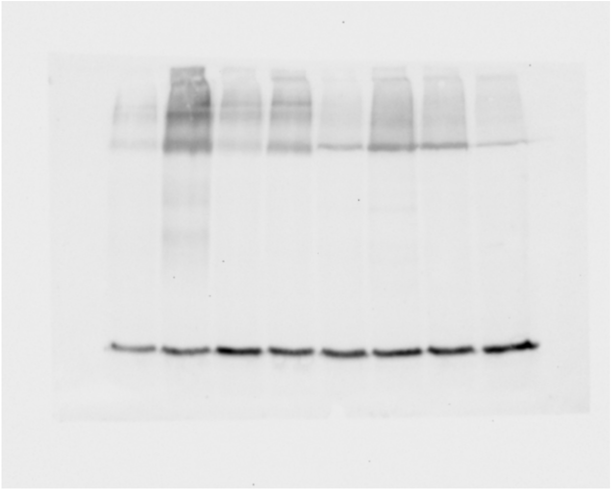

antiGFP + anti-actin

Full size images for hybridations of *gpdA* driven *enaA-gfp* chimera
See Figure 7 panels A and B, and legend for lanes information.

Panel
WT background

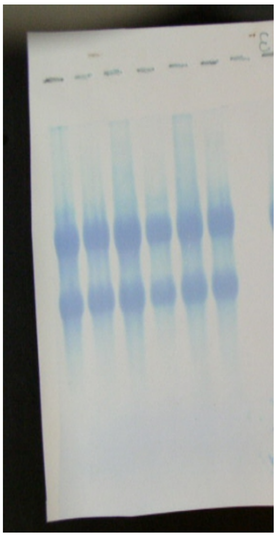

rRNA

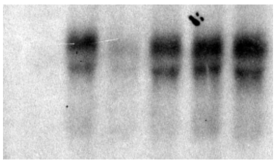

probe *enaA*

Panel B,
palA1 background

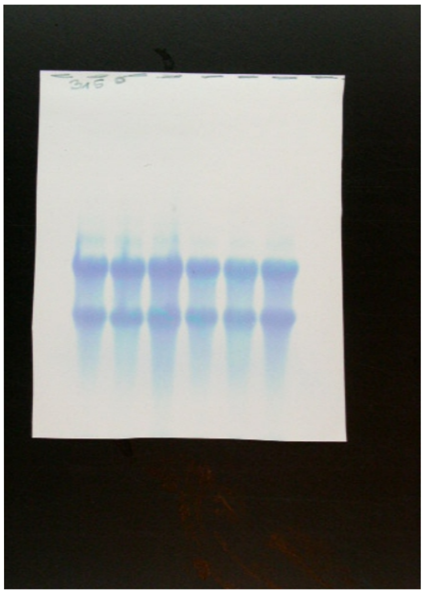

rRNA

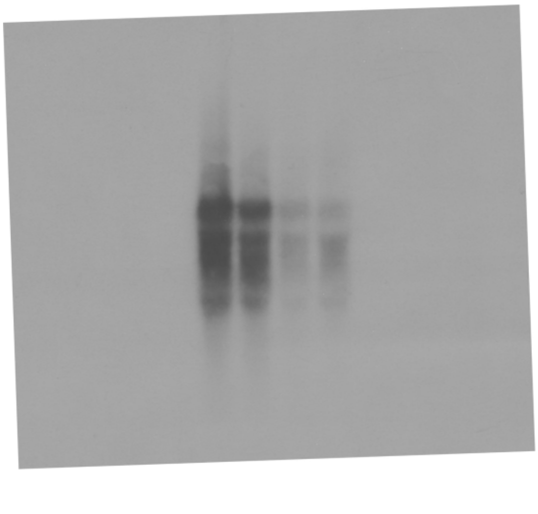

probe *enaA*
